# Epigenetic Landscape of H3K27ac, H3K27me3, H3K4me1, and H3K4me3 Marks in Channel Catfish Following Βeta Glucan Exposure

**DOI:** 10.3390/ijms27146282

**Published:** 2026-07-15

**Authors:** Samah Attia Algharib, Larry Hanson, Lora Petrie-Hanson

**Affiliations:** 1 Department of Comparative Biomedical Sciences, College of Veterinary Medicine, Mississippi State University, 240 Wise Center Drive, Starkville, MS 39762, USA; saa403@msstate.edu (S.A.A.); lah3@msstate.edu (L.H.); 2 Department of Clinical Pathology, Faculty of Veterinary Medicine, Benha University, Moshtohor 13736, Egypt

**Keywords:** trained immunity, epigenetic changes, chromatin landscape, β-glucan, channel catfish

## Abstract

Epigenetic regulation plays a central role in shaping innate immune responses following immunostimulant exposure. In this study, we characterized the epigenetic landscape of four histone modifications, H3K27ac, H3K27me3, H3K4me1, and H3K4me3, in the anterior kidney of *Ictalurus punctatus* (channel catfish) following β-glucan exposure. Using chromatin immunoprecipitation sequencing (ChIP-seq), we generated chromatin state maps and examined the regulatory potential of these histone marks in relation to transcriptional responses. Genes showing increased expression associated with H3K4me3 up-peaks included regulators of metabolism, signaling, pathogen recognition, and immune regulation, while H3K27ac and H3K4me1 marks were linked to genes involved in apoptosis, cytoskeletal dynamics, autophagy, cell adhesion, and stress responses. In contrast, H3K27me3-associated gene repression appeared to fine-tune immune activation by selectively maintaining transcriptional restraint at specific loci. Further transcriptional regulatory-related genes were significantly enriched, suggesting metabolic adaptation following β-glucan exposure. Collectively, these findings define the epigenetic landscape of key histone marks in channel catfish following β-glucan exposure and reveal coordinated chromatin and transcriptional remodeling, consistent with mechanisms of trained immunity. This work provides novel insight into conserved epigenetic features of innate immune memory in teleost fish and supports the use of β-glucan as a functional immunomodulator in aquaculture.

## 1. Introduction

One of the key molecular mechanisms underlying trained immunity is epigenetic modification, particularly changes in histone marks that alter chromatin accessibility and transcriptional potential without modifying the DNA sequence [1]. Epigenetic reprogramming enables immune cells to retain a “memory” of prior stimulation, leading to faster, more robust gene expression upon secondary challenge. Specific histone modifications, including acetylation and methylation at certain lysine residues of histone H3, are well known to control the activation or repression of immune-related genes [2]. Epigenetic regulation plays a critical role in modulating immune responses in vertebrates, including teleost fish. Specific histone marks are associated with either gene activation or repression and fine-tune the expression of immune-related genes during host–pathogen interactions. H3K27ac, H3K27me3, H3K4me1, and H3K4me3 are of particular interest in immune regulation [3]. H3K27ac (Histone H3 lysine 27 acetylation) is an activation mark enriched at active enhancers and promoters, associated with open chromatin and high transcriptional activity. H3K27me3 (Histone H3 lysine 27 trimethylation) is a repressive mark deposited by the polycomb repressive complex 2 (PRC2) and linked to transcriptional silencing and maintenance of cell identity [4]. H3K4me1 (Histone H3 lysine 4 monomethylation) is a hallmark of poised or active enhancers, often marking regulatory elements that can be rapidly activated upon stimulation. Additionally, H3K4me3 (Histone H3 lysine 4 trimethylation) is an activation mark enriched at the promoters of actively transcribed genes [5,6]. The histone modifications examined in this study were chosen because of their known roles as indicators of active chromatin states linked to trained immunity. We acknowledge that other marks, including H3K9me3, H3K36me3, H3K4me2, or H3ac, could offer additional insights into chromatin regulation and should be explored in future research.

**Figure 1:**
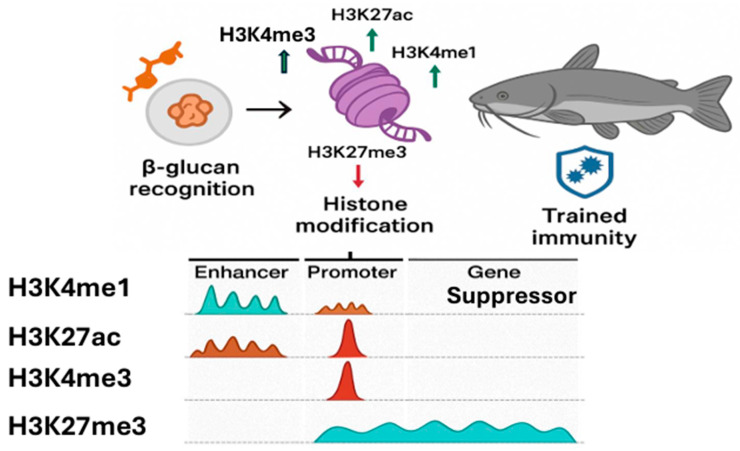
Β Glucan-induced remodeling of the epigenetic landscape in channel catfish innate immune cells. Exposure to β-glucan alters the distribution of key histone modifications (H3K27ac, H3K27me3, H3K4me1, and H3K4me3) in the anterior kidney of *Ictalurus punctatus*, resulting in coordinated changes in chromatin accessibility and gene regulation. Integration of ChIP-seq and transcriptomic analyses reveals selective activation and repression of genes involved in innate immune signaling, metabolism, cytoskeletal organization, and cellular stress responses. Pathway enrichment (KEGG and GO) and gene set enrichment analysis (GSEA) indicate modulation of metabolic and transcriptional pathways, consistent with functional reprogramming of innate immune cells and trained immunity following β-glucan exposure.

Β-glucan, a polysaccharide derived from fungal and yeast cell walls, induces trained immunity [7]. Β-glucan is widely used as an immunostimulant in aquaculture and has been reported to induce trained immunity and enhance disease resistance in fish [8]. In mammals, β-glucan exerts its effects by modulating innate immune pathways, promoting cytokine production, and influencing leukocyte activity [9]. When β-glucan-treated mice were administered lipopolysaccharide (LPS) to induce systemic inflammation, their cells produced more cytokines, and this effect lasted for up to a month [10]. While β-glucan-induced trained immunity is well understood in mammals, with LPS as a secondary stimulus, it is important to recognize that LPS recognition and signaling vary between mammals and teleost fish. Thus, the murine model is used here to illustrate the overall idea of an increased secondary response after β-glucan priming, rather than to suggest exact mechanistic similarity in channel catfish. The development of ‘central’ innate immune memory involves epigenetic modification of hematopoietic stem cells in the bone marrow (or its equivalent) and produces innate immune cells with memory-like characteristics. These cells subsequently traffic to peripheral tissues to produce peripheral memory [11]. Administering β-glucan often stimulates the immune system and can boost resistance to bacterial and viral illnesses [12]. Compared to traditional live attenuated or inactivated vaccines, β-glucan provides several benefits as an immunostimulant. It promotes trained immunity, offering broad non-specific protection against various pathogens without the need for antigen-specific priming. Exposure to β-glucan alters the distribution of key histone modifications (H3K27ac, H3K27me3, H3K4me1, and H3K4me3), resulting in coordinated changes in chromatin accessibility and gene regulation (Figure 1). Generally well tolerated, β-glucan can be administered via multiple methods such as feed or immersion, making it especially effective during early life stages when adaptive immunity is still developing. However, unlike standard vaccines, β-glucan does not elicit pathogen-specific adaptive immunity and should be considered a complementary approach rather than a substitute for vaccination.

There are distinct systems responsible for β-glucan identification and/or downstream signaling in both mammalian vertebrates [13] and invertebrates [14]. However, the precise mechanisms underlying the induced effects in teleost fish are not well understood because a dectin-1 homologue has not been identified. Instead, β-glucan recognition is thought to be mediated by a C-type lectin receptor (CLR). The identification of multiple candidate β-glucan receptors and the typical signaling pathway associated with CLR activation in a model of gene expression profile regulation suggested that signaling by a member of the CLR family may be responsible for the immunomodulatory effects of β-glucan in carp macrophages [15]. Mapping genome-wide changes in histone marks following β-glucan treatment highlights chromatin landscape shifts associated with immune activation or repression, as reviewed in Rodrigues et al. [16]. These associations also contribute to a broader understanding of trained immunity in fish, and pathway analyses indicated that these epigenetic modifications were linked with upregulation of essential immune pathways, including actin cytoskeleton regulation, MAPK signaling, endocytosis, focal adhesion, cell adhesion molecules, Toll-like receptor signaling, phagosomes, AGE-RAGE signaling, and reduced Wnt signaling via decreased H3K27me3 [8]. Building on this foundation, the present study investigated the chromatin landscape in greater depth to define how specific histone modifications regulate immune pathways in fish. By integrating chromatin immunoprecipitation sequencing with transcriptomic analysis, this project seeks to clarify the epigenetic mechanisms underlying trained immunity in a teleost model and to expand understanding of innate immune memory beyond mammalian systems.

## 2. Results and Discussion

### 2.1. Sequencing Quality and Data Overview

**Figure 2:**
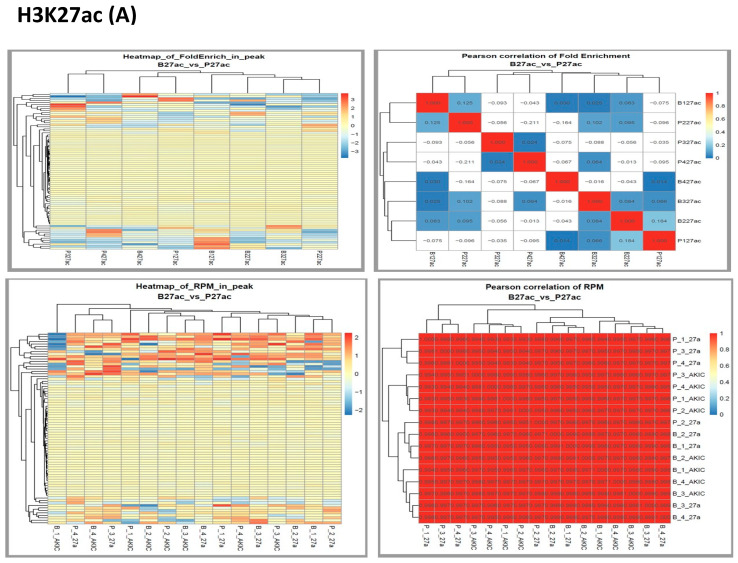

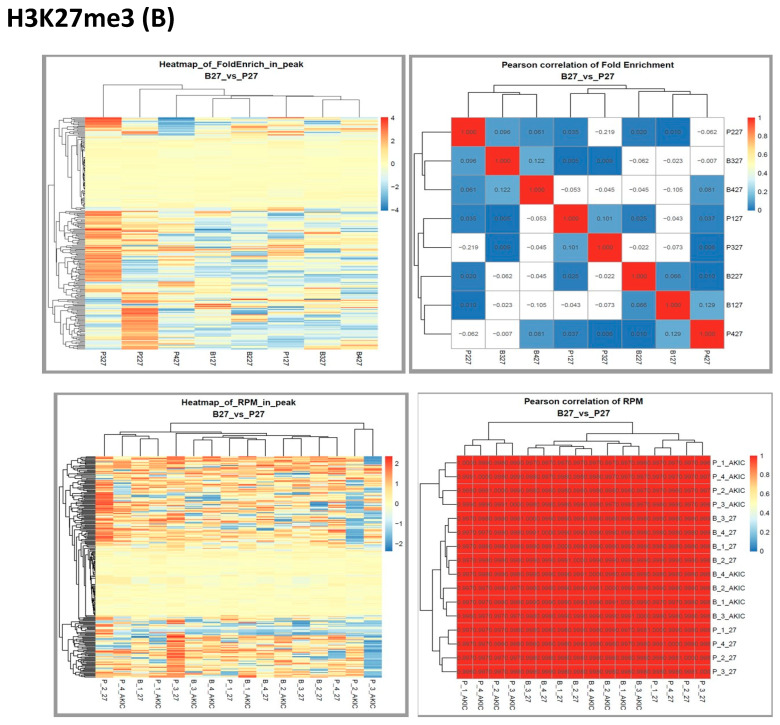

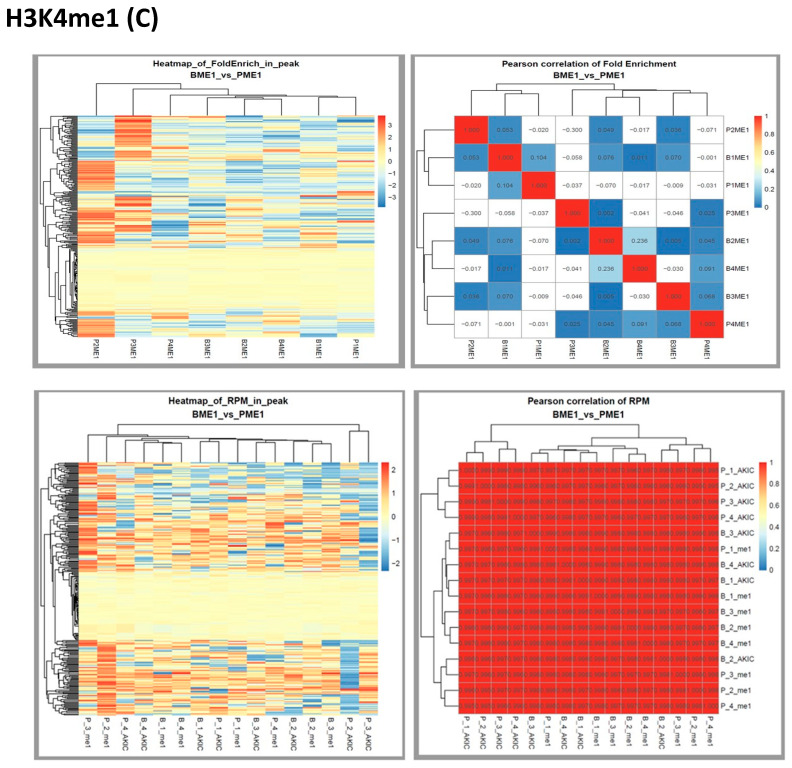

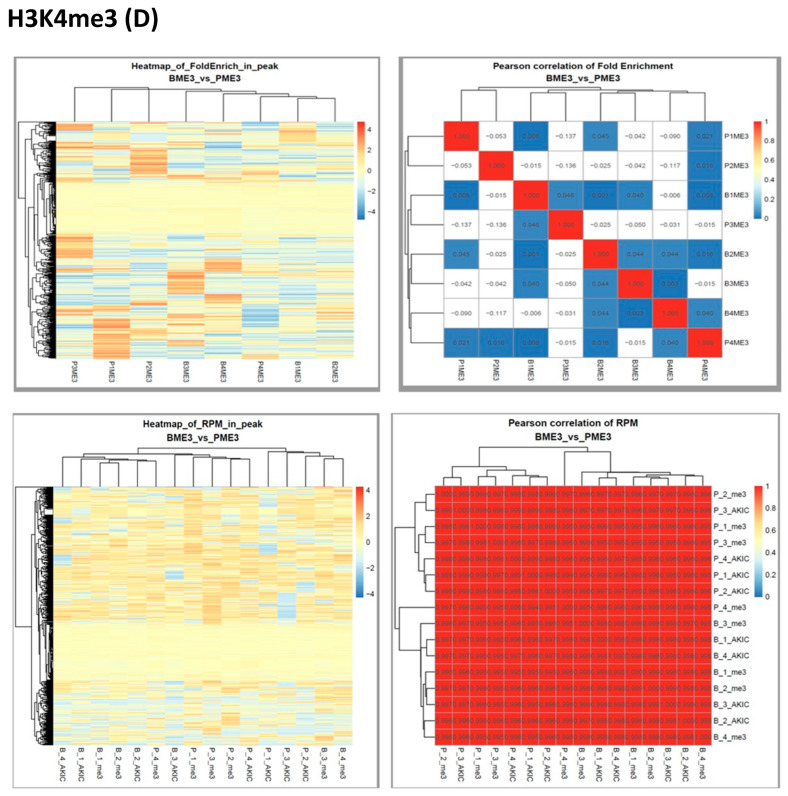
Heat maps and Pearson correlations of fold enrichment and RPM in the peaks of H3K27ac (**A**), H3K27me3 (**B**), H3K4me1 (**C**), and H3K4me3 (**D**). Low correlations and heterogeneous clustering of fold enrichments indicate that β-glucan induces a heterogeneous chromatin response, reflecting a spectrum of epigenetic modifications.

To further interrogate the epigenetic reprogramming underlying β-glucan-induced trained immunity previously associated with enhanced innate immune function, we performed an expanded analysis of histone modification landscapes from the previously described dataset [8]. All histone marks produced well-defined genome-wide peaks, indicating successful immunoprecipitation and sufficient signal enrichment for downstream analysis. Heat maps of normalized read coverage (Figure 2) showed strong clustering of biological replicates at the RPM level, and Pearson correlation analysis of RPM values confirmed high sequencing quality, library integrity, and reproducibility across β-glucan-treated samples. Although saline controls showed slightly lower correlations, the values remained within acceptable quality-control thresholds, indicating adequate sequencing performance. Hierarchical clustering of RPM heatmaps further emphasized local differences in peak intensities despite high global similarity. The consistently low Pearson correlations (<0.4) for fold-enrichment comparisons suggest substantial variability in chromatin profiles across samples. These values might reflect biological differences caused by β-glucan-induced chromatin remodeling, but they could also be influenced by technical variability among replicates. According to standard ChIP-seq quality criteria, low correlation can arise from factors such as differences in immunoprecipitation efficiency or sequencing depth. Nevertheless, several aspects of our study indicate the presence of meaningful biological variation: fish were sampled from multiple tanks, reducing the risk of tank-specific bias. Replicates, formed by pooling samples from several individuals, help lessen the impact of individual outliers. Consistent patterns in pathway enrichment and gene-level analyses further support the existence of biological signals despite the observed variability.

Although RPM-based analyses confirmed strong technical reproducibility and global similarity across libraries, fold-enrichment metrics revealed treatment-specific remodeling events that varied considerably among individuals. Heatmap patterns supported this interpretation, showing distinct enrichment signatures between treatment groups but limited clustering of individual fish. This lack of tight grouping may reflect biological heterogeneity in the anterior kidney, where differences in cell-type composition or immune activation states could influence chromatin responses. Alternatively, β-glucan may elicit individualized epigenetic trajectories, consistent with the complexity of innate immune modulation in teleosts.

These findings highlight the importance of distinguishing between global sequencing consistency (as captured by RPM correlations) and locus-specific regulatory dynamics (as captured by fold-enrichment analyses). The low correlations and heterogeneous clustering indicate that β-glucan does not induce a uniform chromatin response, but rather a spectrum of epigenetic changes that may contribute to variability in immune function. Although some contribution from mixed-cell averaging cannot be excluded, the observed heterogeneity likely reflects biologically meaningful diversity among leukocyte populations. Future single-cell chromatin profiling and cell-type-specific assays will be valuable for resolving the relative contributions of these factors and further defining the cellular basis of trained immunity in fish.

### 2.2. ChIP-Seq Peak Calling and Global Chromatin Landscape

**Figure 3:**
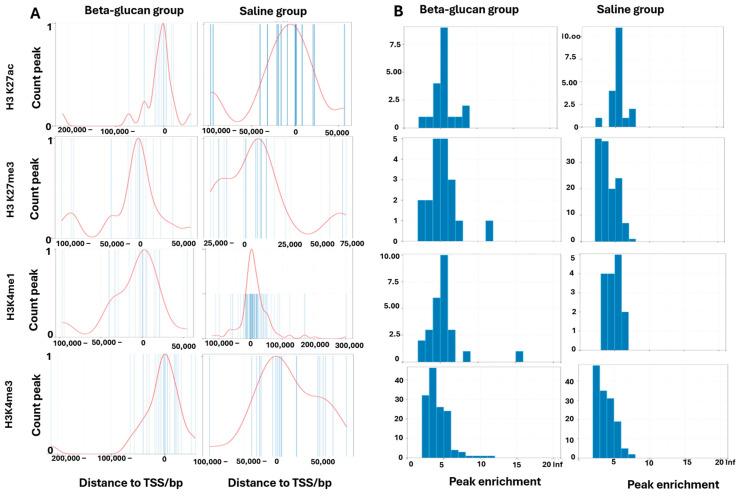
Distributions of peak-transcription start site (TSS) distances (**A**), and peak fold-enrichment distributions (**B**), of H3K27ac, H3K27me3, H3K4me1, and H3K4me3.

Peak calling using MACS2 identified high-confidence regions across all samples. Differential peak analysis using DiffBind (FDR < 0.05) identified peaks significantly enriched following β-glucan exposure relative to saline controls. These differential peaks were distributed across promoter regions, gene bodies, and intergenic elements, with enrichment at loci associated with immune regulation and metabolic pathways (Supplementary Tables S1 and S2). Peak-to-transcription start site (TSS) distance analysis (Figure 3A) revealed distinct distribution patterns for each histone modification. Enrichment of H3K27me3 peaks around the TSS suggests a regulatory role in transcriptional silencing, consistent with PRC2-mediated deposition [17]. In contrast, H3K27ac peaks were observed both upstream and downstream of the TSS, suggesting activity at promoter and enhancer regions. H3K4me3 showed enrichment at the TSS, consistent with transcriptionally active promoters, whereas H3K4me1 exhibited a broader distribution, supporting its role in distal enhancer regulation. These patterns align with previously reported promoter-centric distributions for H3K27ac, H3K27me3, and H3K4me3, and mixed distributions for H3K4me1 [18]. In our study, the contrasting distributions of H3K4me1 and H3K4me3 highlight their complementary roles in gene regulation.

Analysis of peak q-value distributions further emphasized functional differences among histone marks (Figure 3B). H3K27ac and H3K4me3 displayed high −log10 (q-value) peaks, indicating strong enrichment of active regulatory regions, whereas H3K27me3 exhibited broader, lower-significance distributions typical of repressive chromatin. H3K4me1 showed a broad peak distribution, suggesting enhancer-associated activity.

### 2.3. Transcription Factor Motif Enrichment

**Figure 4:**
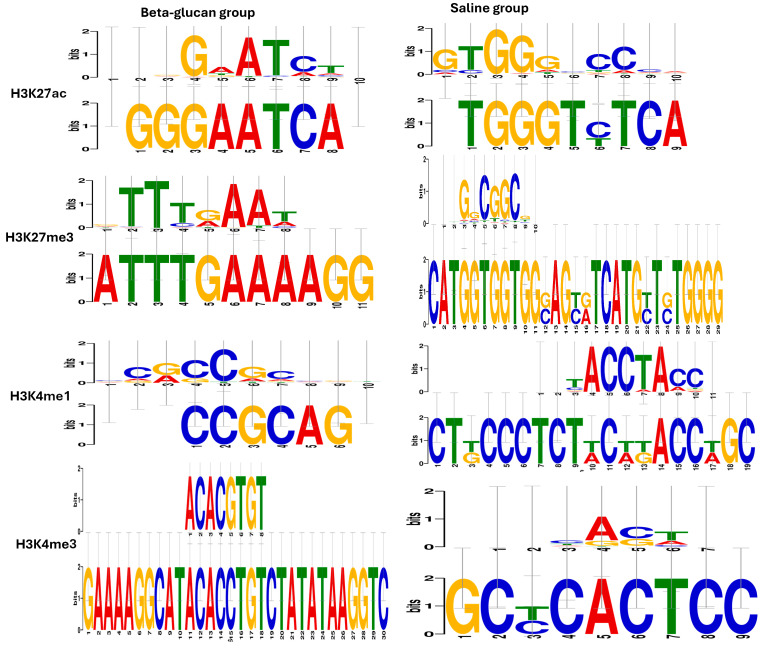
MEME–TOMTOM motif annotation of ChIP-seq peaks. De novo motif discovery and annotation were performed on differentially enriched ChIP-seq peak regions for H3K27ac, H3K27me3, H3K4me1, and H3K4me3 in β-glucan-treated versus saline-treated channel catfish. Enriched motifs correspond to putative transcription factor binding sites associated with immune signaling, metabolic regulation, and transcriptional control.

Motif discovery analysis was performed on ChIP-seq peak regions as described in Ma et al. [19], identifying conserved sequence motifs enriched within regulatory regions associated with each histone modification. These motifs represent putative transcription factor and co-regulator binding sites (Figure 4). Within H3K27ac-enriched regions, the motif GGGAATCA was identified most prominently. This sequence is consistent with binding motifs recognized by members of the NF-κB transcription factor family, which regulate genes involved in inflammation, cytokine production, and innate immune signaling [20]. The enrichment of NF-κB-like motifs within H3K27ac peaks suggests that β-glucan exposure promotes activation of enhancer regions associated with immune-responsive genes. Motifs enriched in H3K27me3-associated regions included GAAA repeats, resembling interferon regulatory factors (IRF) binding motifs and interferon-stimulated response element (ISRE)-like sequences (GAAANNGAAA). However, the presence of transcription factor-binding motifs alone does not indicate actual binding or regulatory activity, particularly in regions marked by H3K27me3, which is generally associated with transcriptional repression. Notably, RNA-seq analysis revealed downregulation of IRF family members (irf4 and interferon stimulated exonuclease gene 20-like 2 (unpublished data) following β-glucan treatment. This finding suggests that IRF-mediated regulatory activity may be reduced under these conditions, consistent with the enrichment of these motifs in regions associated with repressive chromatin marks.

Because H3K27me3 is generally associated with transcriptional repression, the presence of IRF-related motifs in these regions may indicate poised or selectively repressed immune regulatory elements, consistent with the fine-tuning of immune activation following β-glucan exposure. Members of the IRF family play central roles in antiviral defense, interferon signaling, and immune regulation [21].

For H3K4me1-enriched regions, the CCCAG motif was frequently observed. This motif is like consensus sequences recognized by NF-κB and other immune-related transcription factors, supporting the association of H3K4me1-marked enhancer regions with immune gene regulation. H3K4me1 is commonly associated with active or primed enhancers, and enrichment of immune-related motifs within these regions suggests enhancer priming at loci involved in innate immune responses.

Although NF-κB and IRF/ISRE motifs were not always the most statistically significant, they were among the notably enriched motifs and were chosen for further study because of their known importance in innate immune regulation. These transcription factors play key roles in pathways like Toll-like receptor and interferon signaling, which are closely linked to β-glucan-induced trained immunity. Their selection was based on both biological significance and statistical enrichment.

Analysis of H3K4me3-enriched regions identified repeated occurrences of the ACACGTGT sequence, corresponding to an extended E-box motif (CACGTG). E-box motifs are recognized by basic helix–loop–helix transcription factors, including MYC, which regulates genes involved in cell proliferation, metabolism, and transcriptional amplification [22]. While MYC is not a primary immune-specific transcription factor, its presence within H3K4me3-marked promoters may reflect increased transcriptional activity and metabolic support required for immune cell activation and proliferation following β-glucan exposure.

Collectively, these motif enrichment patterns suggest that β-glucan-induced epigenetic remodeling facilitates selective engagement of transcriptional programs mediated by immune- and metabolism-associated transcription factors. The distribution of transcription factor motifs across activating and repressive histone marks supports a model in which chromatin remodeling enables both the activation and restraint of immune pathways, thereby contributing to the functional reprogramming of innate immune cells.

### 2.4. Genomic Annotation and Functional Enrichment Reveal Epigenetic Reprogramming of Immune and Metabolic Pathways

#### 2.4.1. Chromatin Landscape Analysis Correlated with KEGG Pathways

**Figure 5:**
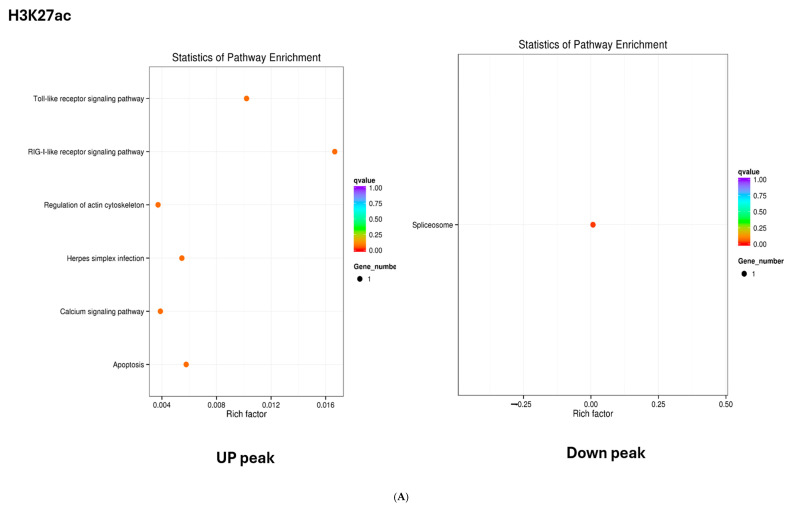

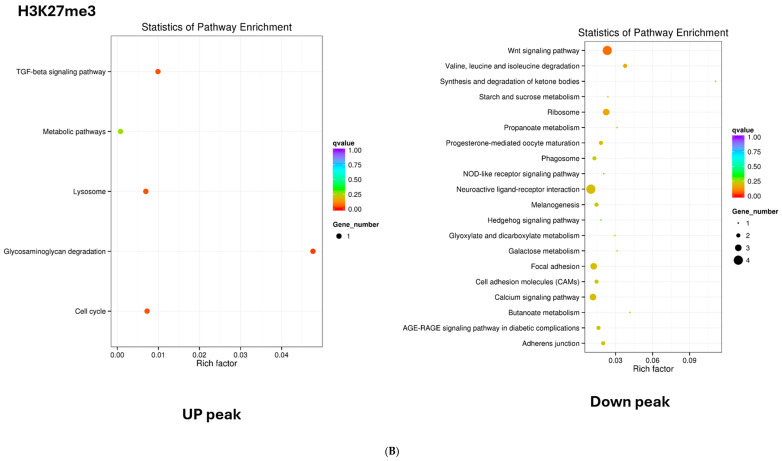

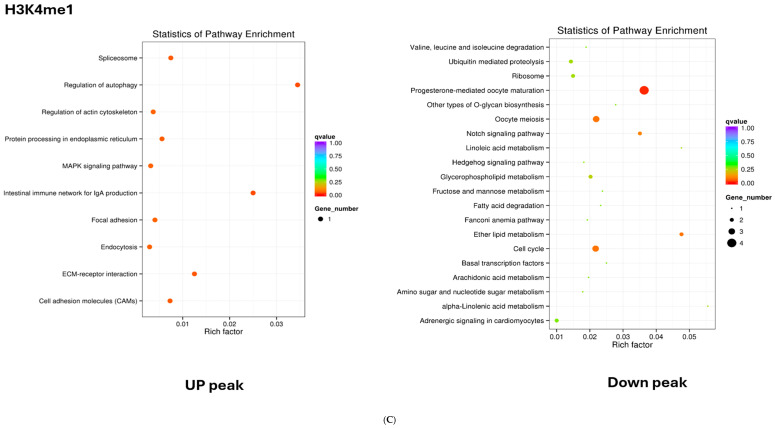

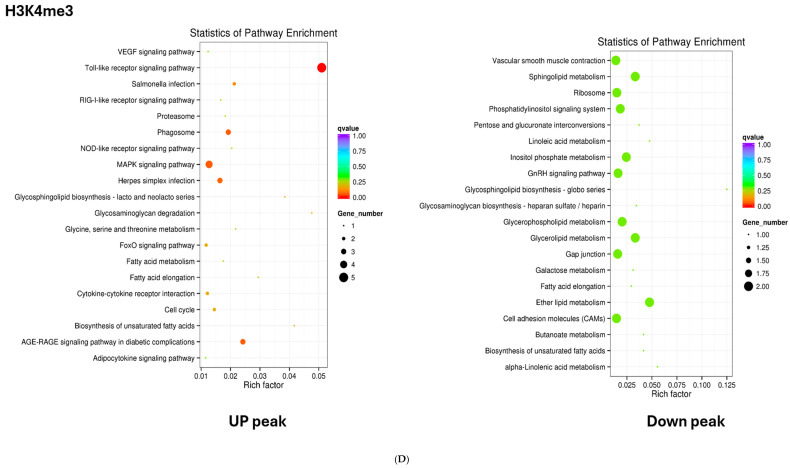
KEGG pathway analyses of H3K27 and H3K4 reconfigurations. In these scatter plots, enrichment is shown by the rich factor, q-value, and gene counts enriched in the pathway. The rich factor is the ratio of differentially expressed gene counts to the total number of genes in the pathway. The higher the rich factor, the higher the degree of enrichment. The q value ranges from 0 to 1 and represents the adjusted *p*-value after multiple hypothesis testing. A low q-value indicates greater enrichment significance. (**A**) Pathways that showed H3K27ac modifications at associated gene loci. (**B**) Pathways that showed H3K27me3 modifications at associated gene loci. (**C**) Pathways that showed H3K4me1 modifications at associated gene loci. (**D**) Pathways that showed H3K4me3 modifications at associated gene loci. The *y*-axis shows the name of the pathway; the *x*-axis shows the rich factor. Dot size represents the number of different genes whose peaks overlap in this pathway, and the color indicates the q-value.

KEGG pathway analysis revealed histone mark-specific enrichment patterns following β-glucan exposure (Figure 5). Up-peaks associated with H3K27ac were enriched in pathways including Toll-like receptor signaling, RIG-I-like receptor signaling, cytoskeletal regulation, apoptosis, and phagocytosis. In contrast, pathways associated with H3K27ac down-peaks were related to transcriptional repression and RNA processing. Increased H3K27me3 enrichment (up-peaks) in β-glucan-treated fish was associated with pathways including TGF-β signaling, lysosomal activity, and cell cycle regulation. Pathways associated with decreased H3K27me3 enrichment included signaling pathways such as Wnt, Hedgehog, and calcium signaling. H3K4me1 up-peaks were enriched in pathways related to immune response, signal transduction, and cytoskeletal organization, whereas down-peaks were associated with metabolic and developmental pathways. H3K4me3 up-peaks showed enrichment in Toll-like receptor signaling and other immune-related pathways, while down-peaks were associated with lipid signaling and protein synthesis pathways. These results demonstrate distinct pathway-level associations for each histone modification following β-glucan exposure.

The enrichment of immune-related pathways across multiple histone marks is consistent with enhanced innate immune activity following β-glucan exposure. Pathways related to pathogen recognition, phagocytosis, and cytoskeletal regulation suggest coordinated changes in immune cell function and structure. The involvement of metabolic and signaling pathways further aligns with the concept of immune cell reprogramming. H3K27me3 is a Polycomb-mediated repressive mark that suppresses a broad spectrum of developmental, metabolic, and signaling pathways [23]. This broad repression contrasts with the more targeted transcriptional downregulation observed for H3K27ac, where only spliceosome-related pathways were consistently reduced.

H3K4me1, a modification associated with enhancer regions and regulatory plasticity [24] showed enrichment at peaks in pathways related to immune responses, signal transduction, and cytoskeletal organization, suggesting heightened enhancer activity and cellular activation. Conversely, down-peaks of H3K4me1 were enriched in metabolic, developmental, and transcriptional regulation pathways, indicating shifts in cellular priorities or differentiation states. H3K4me3, a hallmark of active promoters, showed strong enrichment in Toll-like receptor signaling and in pathways associated with inflammation, immune defense, and angiogenesis, indicating enhanced transcriptional initiation of genes central to host defense and vascular remodeling. These KEGG findings are consistent with previous reports in crustaceans and vertebrates, demonstrating that dietary β-glucan induces enrichment of metabolic pathways, phagosomes, focal adhesion, adherens junctions, PI3K–Akt signaling, MAPK signaling, Rap1 signaling, calcium signaling, and apoptosis [25,26,27]. Collectively, KEGG analysis indicates that histone remodeling following β-glucan exposure preferentially targets immune recognition, phagocytosis, cytoskeletal dynamics, and metabolic signaling. However, these observations are based on pathway-level associations derived from ChIP-seq data and should be interpreted cautiously, as they do not establish direct functional outcomes or causal mechanisms. Collectively, these findings suggest that β-glucan exposure is associated with coordinated chromatin changes in pathways relevant to immune and metabolic processes, although further functional validation is required to confirm their biological significance.

#### 2.4.2. GO Term Network Analysis Identified Immune-Related Terms

**Figure 6:**
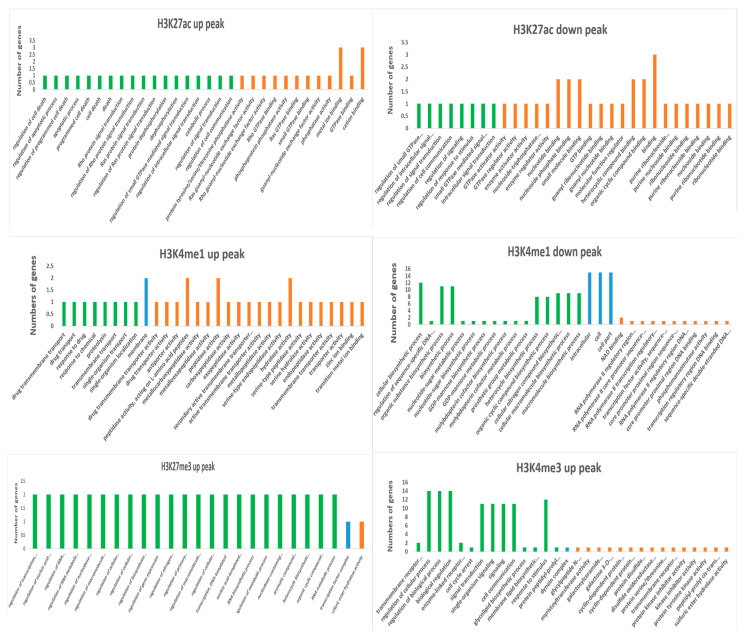
GO enrichment of H3K27 and H3K4 reconfigurations generated in this study. Column with green color (biological process), orange color (molecular function), and blue color (cellular component).

To refine the functional interpretation of KEGG-identified pathways, GO enrichment analyses were performed on genes overlapping with histone-modified regions (Figure 6). GO terms with *p*-values < 0.05 were considered significant and categorized into molecular function, biological process, and cellular component.

H3K27ac up-peaks yielded 21 significant GO terms, with the most enriched molecular function being protein tyrosine/serine/threonine phosphatase activity (GO:0008138; *p* = 0.024835), supporting enhanced signaling regulation during immune activation. H3K27me3 upregulation was associated with 33 significant GO terms, dominated by sulfuric ester hydrolase activity (GO:0008484; *p* = 0.0056792) and transcription factor complex assembly (GO:0005667; *p* = 0.009922), consistent with repression and modulation of transcriptional control mechanisms.

H3K4me1 up-peaks were associated with 43 significant GO terms, with drug transmembrane transport (GO:0006855; *p* = 0.0011379) as the most enriched, reflecting increased membrane transport and cellular exchange. In contrast, H3K4me1 down-peaks showed 72 significant GO terms, led by NAD binding (GO:0051287; *p* = 0.004827). The genes ctbp2 and ric8 contributed to this enrichment and appeared across multiple cofactor- and small-molecule-binding pathways, along with gnai2b, pnck, raver1, trim23, and atp1a3a. In the cellular component category, intracellular parts and intracellular organelles contained the highest number of genes, while biological process terms were dominated by cellular and organic substance biosynthetic processes, indicating broad metabolic regulation.

For H3K4me3, the most enriched GO term was the transmembrane receptor protein serine/threonine kinase signaling pathway (GO:0007178; *p* = 0.0044501), indicating enhanced signal transduction. Upregulated H3K4me3-associated GO terms further indicated increased biosynthesis, protein regulation, membrane remodeling, and cell-surface modification, including enrichment for glycosyltransferase complexes and outer-cell orientation [28,29]. Together, GO enrichment analyses suggest that enhancer and promoter activation drives dynamic immune signaling and metabolic adaptation, whereas repressive marks fine-tune transcriptional restraint during immune reprogramming [30].

### 2.5. Gene Set Enrichment Analysis (GSEA)

Although multiple GO terms were significantly enriched, the detailed interpretation focused on transcription factor binding (tfb) (GO:0008134) because this term directly reflects the activation of key transcriptional co-regulators and DNA-binding factors that drive chromatin remodeling and metabolic gene expression, both essential for establishing β-glucan-induced trained immunity.

**Figure 7:**
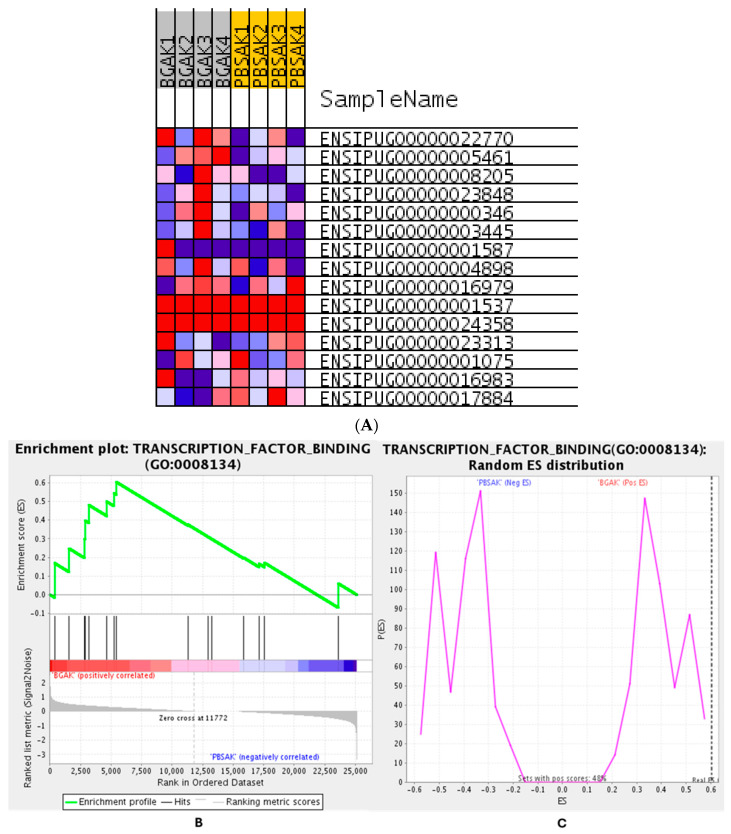
Transcription factor binding (GO:0008134) Blue-Pink O’ Gram in the space of the analyzed gene set, (**A**). Enrichment plot profile of the running ES score and positions of gene set members on the rank-ordered list, (**B**). Random ES distribution, gene set null distribution of ES, (**C**).

We analyzed 15 genes (Figure 7A), of which 8 were identified as core enrichment genes: crtc3, crtc1b, ncoa2, ncoa3, foxa3, and ncoa1. These genes consistently exhibited positive rank metric and enrichment scores, indicating coordinated upregulation (Table 1 and Figure 7). The enrichment of tfb (GO:0008134) in the GSEA highlights a crucial regulatory layer in the metabolic reprogramming underlying β-glucan-induced trained immunity. When exposed to β-glucan, innate immune cells initiate a coordinated transcriptional response driven by key transcription factors such as HIF-1α, NF-κB, AP-1, and the STAT family, which bind to regulatory regions of glycolysis, cholesterol synthesis, and mitochondrial function. This enrichment suggests that trained monocytes and macrophages rely on increased access and activity at these transcription factor binding sites to sustain a shift towards aerobic glycolysis, fumarate buildup, and activation of the mevalonate pathway, metabolic features indicative of trained immunity [31]. This finding supports earlier research showing chromatin remodeling at transcription factor sites is vital for reprogramming inflammatory and metabolic genes during training. Therefore, activation of transcription factor networks serves not only as a response to β-glucan stimulation but also as a foundational mechanism linking metabolic signals with epigenetic memory, enabling a stronger response to secondary challenges. The presence of factors such as crtc3, crtc1b, ncoa2, ncoa3, foxa3, and ncoa1 within this pathway underscores the role of transcriptional co-activators in shaping the metabolic and epigenetic features of trained innate immunity. CRTC proteins act as strong co-activators of CREB, which regulates enzymes involved in glycolysis, mitochondrial function, and stress responses. Their presence indicates enhanced CREB-mediated transcription, promoting the metabolic shift toward aerobic glycolysis characteristic of trained cells [32]. Similarly, NCOA1/2/3, nuclear receptor co-activators, integrate metabolic signals, such as lipid-derived ligands and acetyl-CoA levels, thereby enhancing chromatin accessibility and activating genes involved in cholesterol synthesis, oxidative metabolism, and inflammation that are integral to trained immunity. The inclusion of FOXA3, a pioneer factor that opens tightly packed chromatin, supports the idea that early chromatin remodeling during β-glucan training enables long-term accessibility at loci relevant to metabolism and immunity [33].

Among the core-enriched genes, ENSIPUG00000000346 and ENSIPUG00000003445 lack annotations in the current Ensemble releases, a common feature in non-model genomes such as channel catfish. Their strong enrichment in tfb (GO:0008134) indicates involvement in transcriptional regulation (Figure 7B). Including them suggests they may encode unknown transcription factor partners or chromatin proteins that facilitate transcription and epigenetic changes during β-glucan-induced trained immunity. Because trained immunity depends on activating transcriptional networks that control glycolysis, mitochondria, and inflammation, these genes could be novel regulators linking metabolic signals to transcriptional memory. Their presence highlights the role of unexplored regulatory elements in innate immune training and offers targets for future studies.

### 2.6. Integration of Differentially Expressed Genes and Histone Modifications in Trained Immunity

**Figure 8:**
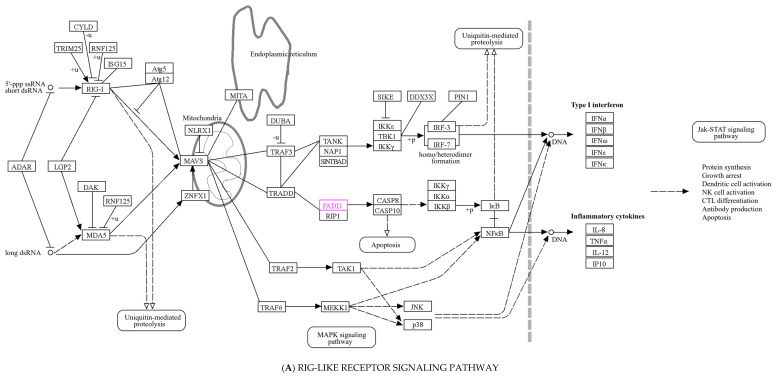

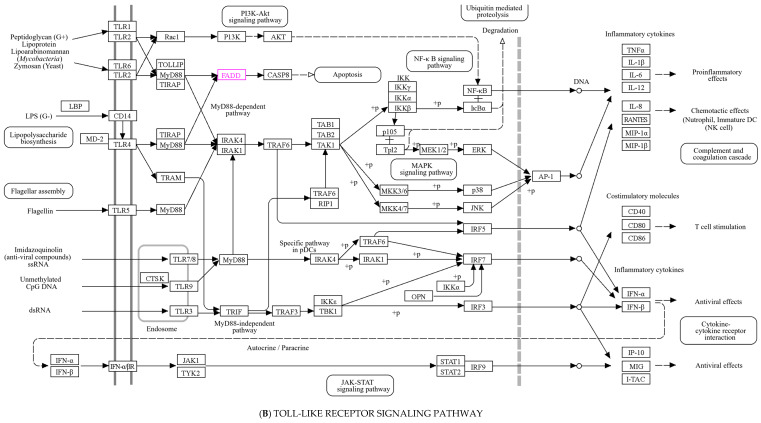

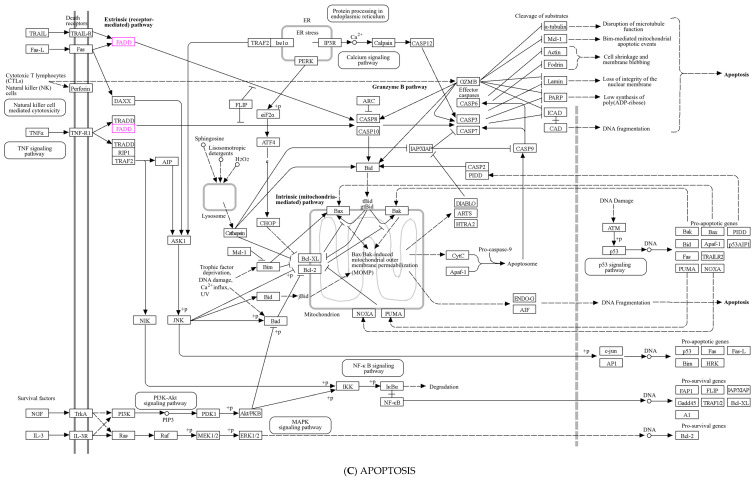
RIG-1-like receptor signaling (**A**), Toll-like receptor signaling (**B**), and apoptosis (**C**) pathways related to *FADD* gene (pink color), adapted from the Kyoto Encyclopedia of Genes and Genomes (KEGG; Ogata et al., [36]).

Genes with an FDR < 0.05 and an absolute log_2_ fold change ≥ 1 were considered significantly differentially expressed. Differential histone modification patterns identified multiple affected gene loci. In total, 718 DEGs were detected in β-glucan-injected fish compared with physiological saline controls, including 397 upregulated and 321 downregulated genes. Integration of DEG profiles with epigenetic data demonstrated that β-glucan induces coordinated remodeling of H3K27ac, H3K27me3, H3K4me1, and H3K4me3 at immune-relevant loci in channel catfish anterior kidney leukocytes (Supplementary Table S3). Genes associated with H3K27ac up-peaks included *fadd*, *arhgef6*, and *si:dkey-28b4*, indicating active transcription of immune signaling genes. *Fadd* exhibited a four-fold increase in both H3K27ac and H3K4me3 and was linked to RIG-I-like receptor signaling, Toll-like receptor signaling, apoptosis and NOD-like receptor signaling (Figure 8). As a central adaptor in death receptor signaling, *fadd* regulates apoptosis and necroptosis while also contributing to NF-κB and MAPK pathway activation, thereby balancing immune activation and resolution [34]. The enrichment of H3K27ac and H3K4me3 at the fadd gene locus indicates increased transcriptional activity; however, this does not necessarily mean an enhanced capacity for apoptosis. In trained immunity, *fadd* might influence innate immune signaling pathways, potentially promoting survival and inflammation instead of solely inducing apoptosis. *Arhgef6*, a cytoskeletal regulator, plays critical roles in immune cell migration and T- and B-cell trafficking [35], supporting enhanced cellular mobilization during trained immunity.

In contrast, increased H3K27me3 enrichment correlated with downregulation of *ids*, *id2a*, and *dp-2*. *IDS* exhibited comparable enrichment for both H3K27me3 and H3K4me3, suggesting a bivalently chromatinized state poised for activation but not fully transcribed [37]. Conversely, genes with reduced H3K27me3 enrichment showed higher expression, including *plcb4*, *ctnnb1*, *cxcr3.2*, *itgb1b*, *hsp90a2*, *rac1*, *hmgcl*, and *odc1*. These findings support the reversibility of polycomb-mediated silencing during trained immunity and highlight the contribution of H3K27me3-regulated genes to immune memory.

Notably, *itgb1b* exhibited a five-fold increase concomitant with decreased H3K27me3, consistent with enhanced cell adhesion, migration, and metabolic support for sustained immune activation [38]. *Plcb4* showed extensive regulation across both H3K27me3 and H3K4me3 and was associated with multiple signaling pathways, including Wnt, calcium, AGE-RAGE, and phosphatidylinositol signaling. While down-peak enrichment suggests resolution of a bivalent domain and transcriptional silencing [39], *plcb4* may be reactivated during trained immunity to support calcium-dependent signaling and activation of the C-type lectin receptor (CLR) pathway. Consistent with this interpretation, manipulation of *plcb4* has been proposed as a therapeutic strategy for immune reprogramming [40].

**Figure 9:**
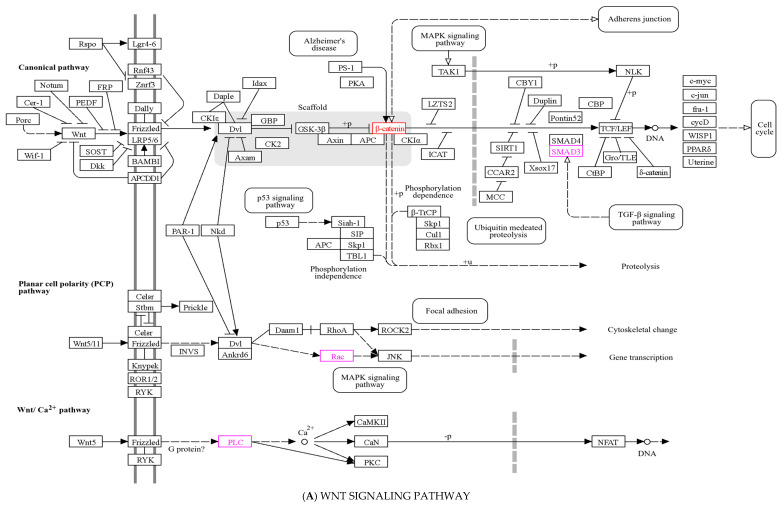

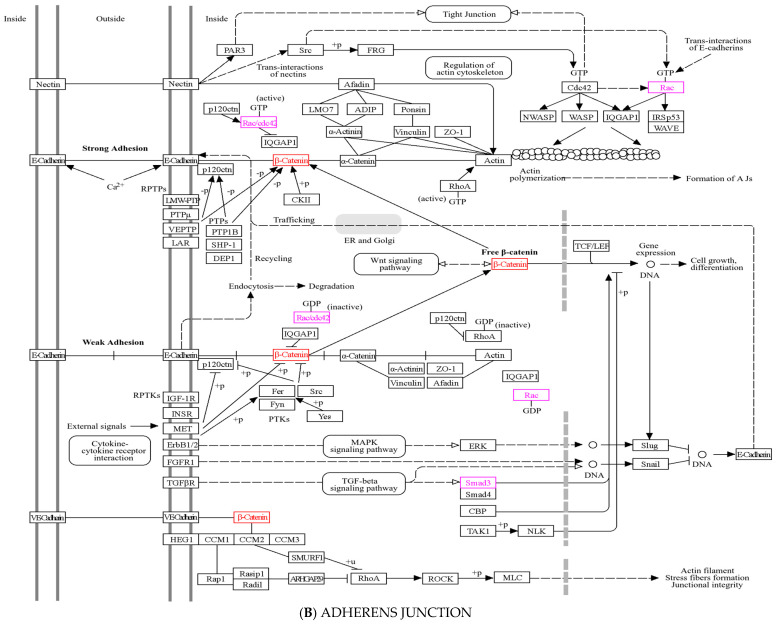

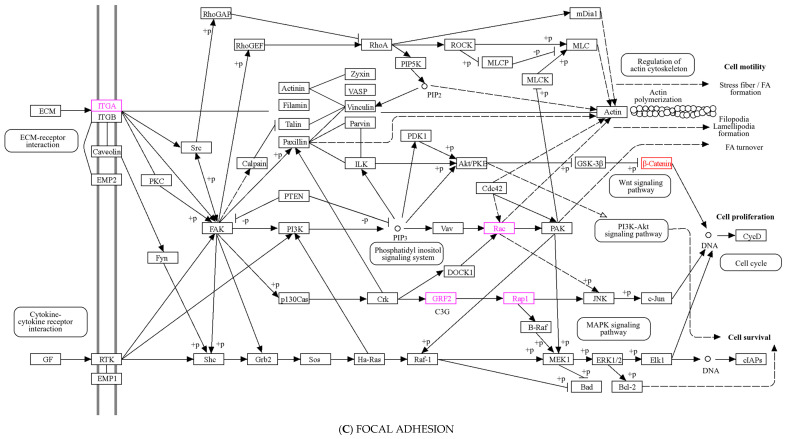
Wnt signaling (**A**), adherens junction (**B**), and focal adhesion (**C**) pathways associated with *catenin* gene (red color) and other genes (pink color) like *Smad3*, *Rac*, and *ITGA*, adapted from the Kyoto Encyclopedia of Genes and Genomes (KEGG; Ogata et al., [36]).

*Catenin*, particularly *β-catenin*, a central effector in the Wnt signaling pathwayIt exhibited increased 5-fold expression alongside a reduction in H3K27me3 enrichment. As H3K27me3 is typically associated with transcriptional repression, its decreased presence at the *β-catenin* locus suggests epigenetic derepression, consistent with enhanced transcriptional activity rather than polycomb-mediated silencing. This finding supports a potential activation of Wnt signaling pathways, adherens junctions, focal adhesions, melanogenesis, and tight junctions. These pathways are crucial for cell adhesion, migration, differentiation, and immune cell communication (Figure 9). Catenin’s role in cell adhesion and transcriptional regulation suggests it may influence CLR-mediated immune responses and the epigenetic flexibility seen in trained immunity [11]. *Rac 1* was increased 5 times, indicating its role in regulating cytoskeletal organization, cell adhesion, migration, and morphological changes [41]. Additionally, there was a 5-fold increase in *hmgcl*, while *hmgcl* is not a direct marker of trained immunity, its role in ketogenesis and leucine metabolism makes it metabolically relevant. Changes in its expression or activity could influence the metabolic state of immune cells, thereby indirectly affecting trained immunity [42]. On the same side, *hsp90aa1*, accompanied by a NOD-like receptor, showed a 3-fold increase. NOD-like receptors detect bacterial peptidoglycan fragments in the cytosol that have escaped from endosomal compartments, triggering NF-κB and MAPK activation, cytokine production, and apoptosis. On the other hand, a distinct set of NOD-like receptors induces caspase-1 activation by assembling multiprotein complexes called inflammasomes. The activation of caspase-1 regulates the maturation of the pro-inflammatory cytokines IL-1β and IL-18, driving pyroptosis [43]. *Hsp90aa1* promotes NLRP3 activation by stabilizing the protein and assisting in its proper folding, which enables the formation of the inflammasome complex. The alpha isoform of *hsp90aa1* contributes to trained immunity by acting as a chaperokine [44]. There are also notable upregulated genes associated with H3K4me1 up peak, including *atga4*, *itga4*, and *hsp70*. These genes support stress adaptation and immune cell mobilization, all of which are vital for trained immunity.

In contrast, H3K4me1 down-peak regions were associated with differential expression of multiple genes, including *pgr*, *gnai2b*, *rad21b*, *ctbp2a*, *fancl*, *gxylt1a*, *taf6*, *phosphomannomutase 1*, *cpt2*, *pi4kaa*, *oprd1*, *cdc16*, *plb1*, *plpp3*, *smad3a*, *rbpjb*, *atp1a3a*, *acsf3*, and *cnot8*. Trained immunity is characterized by chromatin reprogramming rather than uniform enhancer activation, and downregulation of H3K4me1 at specific locations may relieve repression or reflect non-canonical enhancer usage, thereby enabling selective transcription of genes involved in metabolism, signaling, and immune regulation. Many of the genes associated with H3K4me1 down-peaks participate in metabolic reprogramming (e.g., *cpt2*, *acsf3*), transcriptional regulation (e.g., *taf6*, *ctbp2a*), cell cycle control and DNA repair (e.g., *fancl*, *cdc16*), and immune signaling and stress responses (e.g., *smad3a*, *rbpjb*). Regulation of phosphomannomutase therefore provides a mechanistic link between carbohydrate metabolism, glycosylation, and the durable functional changes observed in trained innate immune cells [42,45].

Notably, *gnai2b* and *plpp3* exhibited five-fold increases in expression, while *plb1* and *smad3a* were upregulated six-fold. *gnai2b*, encoding the inhibitory G protein α-subunit Giα2, is a key regulator of GPCR-mediated signaling and leukocyte chemotaxis, influencing immune cell migration, inflammatory tone, and metabolic signaling downstream of chemokine receptors. Altered regulation of *gnai2b* is therefore consistent with enhanced immune cell responsiveness and trafficking during trained immunity. *Plpp3* encodes a lipid phosphate phosphatase that modulates phosphatidic acid and lysophosphatidic acid signaling, thereby influencing membrane dynamics, cell migration, and inflammatory signaling. Together, the repeated expression of these genes in H3K4me1 downregulated regions supports the concept that trained immunity involves selective activation of key signaling and metabolic genes, even in the absence of canonical enhancer marks [46].

In parallel, H3K4me3 up-peak enrichment affected the expression of numerous immune-related genes, including *hacd3*, *ppargc1al*, *itch*, *map3k7ip2*, *map3k8*, *tlr9*, *cdkn1ba*, *rac1*, *tgfb2*, *colec11*, *dync1h1*, *ccr6b*, *b3gnt5a*, *sardh*, *psmd6*, and *mpdz*. Upregulation of these genes, as reflected by H3K4me3 enrichment, underscores their central roles in the epigenetic and functional reprogramming of innate immune cells during trained immunity, spanning metabolism, signal transduction, pathogen recognition, cell migration, and immune regulation.

Metabolic reprogramming is a defining feature of trained immunity, and genes such as *hacd3*, *ppargc1al*, and *sardh* support increased lipid metabolism, mitochondrial regulation, and biosynthetic capacity, thereby enabling immune cells to mount more robust secondary responses [47]. Signal transduction genes, including map3k7ip2, map3k8, rac1, and itch, are integral to CLR- and PRR-mediated signaling cascades that converge on MAPK and NF-κB activation, promoting the production of pro-inflammatory cytokines and antimicrobial effector functions. *Rac1* regulates cytoskeletal remodeling and phagocytosis, linking signaling activation to functional immune responses.

Genes involved in pathogen recognition, such as *tlr9* and *colec11*, enhance innate immune sensing of microbial ligands, often acting synergistically with C-type lectin receptor pathways to broaden pathogen recognition and support the non-specific protection characteristic of trained immunity [48,49]. Additionally, genes associated with cell cycle regulation and migration (*cdkn1ba*, *ccr6b*, *dync1h1*, *mpdz*) facilitate immune cell proliferation and trafficking to sites of inflammation, further amplifying trained responses. Protein modification and degradation genes (*psmd6*, *itch*, *b3gnt5a*) ensure appropriate turnover of signaling components, enabling rapid yet controlled immune activation. Immune regulatory genes such as *tgfb2* contribute to balancing inflammatory activation and resolution, thereby limiting tissue damage and maintaining homeostasis during prolonged immune responses [50].

Although H3K4me3 is classically associated with active promoters, emerging evidence indicates that H3K4me3 downregulation does not universally result in transcriptional silencing [51]. In this study, several genes associated with H3K4me3 down-peaks, including *pla2g4ab*, *plpp1a*, and *skp1*, remained strongly upregulated, revealing non-canonical regulatory mechanisms during trained immunity. *Pla2g4ab* encodes a phospholipase involved in arachidonic acid release and eicosanoid synthesis, supporting membrane remodeling and inflammatory mediator production [52]. *Plpp1a* regulates lipid signaling pathways that influence immune cell migration and membrane dynamics [53], while *Skp1* is a core component of the SCF ubiquitin ligase complex, essential for protein turnover and the fidelity of immune signaling [54]. The regulation of these genes underscores the importance of lipid metabolism, membrane remodeling, and proteostasis in β-glucan-induced trained immunity and highlights the complexity of epigenetic regulation in teleost immune responses.

## 3. Materials and Methods

### 3.1. Fish Acclimation and β-Glucan Exposure

Channel catfish (average weight 80 g, sex not determined) were obtained from a single cohort. Fish were maintained in 30 L tanks, with 10 replicate tanks per treatment group and with 5 fish per tank. Tanks were randomly assigned.

Each tank was supplied with dechlorinated municipal water at approximately 0.2 L/min, with continuous aeration and a temperature of 28 ± 0.2 °C. Fish were fed a commercial catfish diet containing 32% protein at a daily feeding rate of 3% of the total fish biomass/tank. All experimental procedures were approved by the Mississippi State University Institutional Animal Care and Use Committee (MSU IACUC).

Following a one-week acclimation period, fish received intraperitoneal (IP) injections of either physiological saline (0.8 mL/fish) or β-1,3-glucan (Calbiochem, CAS 9012-72-0; derived from *Saccharomyces cerevisiae*) at a dose of 100 µg/g body weight suspended in 0.8 mL saline. The β-glucan dose of 100 µg/g body weight was chosen based on earlier studies in teleost fish showing effective immune stimulation without evident toxicity [8]. In this study, no behavioral issues or increased mortality rates were observed.

Fish were maintained under these conditions for one month, after which anterior kidney tissues were collected for subsequent analysis.

### 3.2. Tissue Collection and Leukocyte Isolation

After one month, the anterior kidney (AK) tissues were collected from four biological replicates of β-glucan-exposed catfish and four biological replicates of the catfish control. Each biological replicate was a pooled sample from 10 fish tissues. Fish were taken from different tanks within the same treatment group to minimize tank-specific effects and better represent the population. These pooled samples were processed individually through chromatin immunoprecipitation and sequencing. Tissues were excised and immediately placed in ice-cold FACS buffer (Hanks’ balanced salt solution lacking calcium and magnesium, supplemented with 0.02% bovine serum albumin). Samples were kept on ice and mechanically dissociated using a Teflon homogenizer (IKA Works, Inc., Wilmington, NC, USA) over a 40 µm cell strainer in FACS buffer. The resulting cell suspension was layered onto a Histopaque 1119 gradient (Sigma-Aldrich, St. Louis, MO, USA; Cat. No. 11191) and centrifuged at 700× *g* for 20 min. Leukocytes were collected from the buffy coat at the gradient interface, washed with 500 µL of FACS buffer, and resuspended at 1 × 10^6^ cells/mL.

### 3.3. Chromatin Preparation and Immunoprecipitation (ChIP)

Chromatin immunoprecipitation was performed on 5 × 10^7^ isolated AK leukocytes using the Zymo-spin ChIP Kit (ZymoResearch D5210, Irvine, CA, USA). The overall chromatin preparation and immunoprecipitation workflow was adapted from established ChIP methodologies with modifications for fish leukocytes and the Zymo-Spin ChIP platform [55]. Cells were fixed in 1% formaldehyde for 7 min, and crosslinking was quenched with 125 mM glycine for 5 min. Nuclei were isolated and lysed in SDS lysis buffer (1% SDS, 10 mM EDTA, 50 mM Tris-HCl, pH 8.1) containing protease inhibitors. Formaldehyde-crosslinked chromatin was fragmented by sonication using a QSONICA Ultrasonic Processor (Model Q115; Qsonica, Newtown, CT, USA) fitted with a CL-18 probe and operated within a 432A sound enclosure. Chromatin samples (500 μL) were kept on ice and sonicated at 25% amplitude for 12 cycles consisting of 15 s of sonication followed by 30 s of cooling. Fragmentation conditions were optimized to generate chromatin fragments ranging from approximately 200 to 300 bp prior to chromatin immunoprecipitation. Chromatin was pre-cleared with Protein A/G magnetic beads for 1 h at 4 °C. Immunoprecipitation was performed overnight at 4 °C using 5–10 μg of the following antibodies: anti-H3K4me1 Ab (Diagenode, Liège, Belgium, Cat. No. CS-037-100), anti-H3K4me3 Ab (Diagenode, Liège, Belgium, Cat. No. pAb-003-050), anti-H3K27ac Ab (Abcam, Cambridge, United Kingdom, Cat. No. ab4729, 0.80 mg/mL), or anti-H3K27me3 (Millipore, Burlington, MA, USA, 07-449). Antibody–chromatin complexes were captured with magnetic beads and sequentially washed with low-salt, high-salt, LiCl, and TE buffers to minimize non-specific binding.

Chromatin was eluted using elution buffer (1% SDS, 0.1 M NaHCO_3_, Sigma-Aldrich, MilliporeSigma, Burlington, MA, USA), and crosslinks were reversed at 65 °C overnight in the presence of 200 mM NaCl. Samples were treated with RNase A and Proteinase K, followed by DNA purification using a column-based ChIP DNA Clean & Concentrator kit (Zymo Research Corporation, Irvine, CA, USA)or phenol–chloroform extraction. DNA concentration and quality were assessed using a Qubit fluorometer (Thermo Fisher Scientific, Waltham, MA, USA) and Agilent 2100 Bioanalyzer (Agilent Technologies, Santa Clara, CA, USA), respectively.

### 3.4. RNA Extraction and Gene Expression Analysis

Total RNA was extracted from isolated leukocytes using the PureLink™ RNA Mini Kit (Invitrogen, Cat. No. 1218018A, Waltham, MA, USA) according to the manufacturer’s instructions. RNA samples were stored at −80 °C until library preparation. To eliminate potential genomic DNA contamination, RNA samples were treated with DNase I prior to downstream applications. RNA integrity was assessed using an Agilent Bioanalyzer (Agilent Technologies, Inc, Santa Clara, CA, USA), with all samples exhibiting RNA integrity numbers (RIN) above 7.0. RNA quality was evaluated using spectrophotometric measurements. All samples showed A260/A280 and A260/A230 ratios within acceptable ranges.

### 3.5. Library Preparation and Sequencing

#### 3.5.1. RNA Sequencing

RNA samples were submitted to Novogene Corporation (Sacramento, CA, USA) for library preparation and sequencing. Quality control was performed using FastQC, followed by adapter trimming and removal of low-quality reads. Strand-specific mRNA libraries were prepared using the TruSeq Stranded mRNA workflow and sequenced on an Illumina platform using paired-end chemistry. Clean reads were aligned to the *Ictalurus punctatus* reference genome using Novogene’s standard RNA-seq pipeline, and uniquely mapped reads were retained for downstream analysis.

#### 3.5.2. ChIP Sequencing

ChIP DNA and corresponding input controls were submitted to Novogene for library preparation and sequencing. Raw reads were assessed using FastQC and processed to remove adapter sequences and low-quality reads. Clean reads were aligned to the *Ictalurus punctatus* reference genome (assembly accession: GCF_001660625.3), which was obtained from the NCBI database (publicly available at https://www.ncbi.nlm.nih.gov/assembly/GCF_001660625.3/ (accessed on 24 June 2021), using BWA, and uniquely mapped reads were identified using a MAPQ threshold of ≥13. PCR duplicates were identified and removed prior to peak calling and downstream analysis. Fragment size distribution and strand cross-correlation metrics (NSC, RSC) were calculated to assess ChIP quality.

### 3.6. Bioinformatic and Statistical Analysis

For RNA-seq analysis, differential gene expression analysis was performed using DESeq2, with significance defined as an adjusted *p*-value (FDR < 0.05). For DESeq2, normalization was performed using the default median-of-ratios method to account for library size and sequencing depth. Genes with low counts were filtered out before analysis; specifically, those with at least 1 per million in a certain number of samples were kept. We also applied DESeq2’s independent filtering to enhance detection power. Log2-fold change estimates were calculated using the default estimator. For ChIP-seq analysis, peak calling was performed using MACS2 with a q-value threshold of 0.05. For MACS2 peak calling, we employed an effective genome size of approximately 7.5 × 10^8^ bp for *Ictalurus punctatus*. Peak calling was performed in either narrow or broad mode, depending on the histone mark: narrow mode for sharp marks such as H3K4me3 and H3K27ac, and broad mode for more diffuse marks such as H3K36me3 and H3K27me3, following standard ChIP-seq protocols.

Differential peak analysis was conducted using MACS2-derived peak sets followed by statistical analysis in R. For functional enrichment analysis, Gene Ontology (GO) and KEGG pathway enrichment analyses were performed using clusterProfiler, with significance defined as FDR < 0.05. For quality control and data validation, all quality control metrics, including read quality, mapping statistics, fragment size distributions, strand cross-correlation analyses, and peak enrichment profiles, were generated by Novogene and used to validate sequencing data prior to downstream analyses.

### 3.7. Integration of ChIP-Seq and Gene Expression Datasets

To define the relationship between chromatin remodeling and transcriptional responses, ChIP-seq and RNA-seq datasets were integrated at the gene level. ChIP-seq peaks identified by MACS2 (q-value < 0.05) were annotated to the nearest gene transcription start site (TSS) using standard genomic annotation tools. Peaks located within promoter regions (defined as ±2 kb of the TSS) and putative enhancer regions were assigned to target genes. For each histone modification (H3K4me1, H3K4me3, H3K27ac, and H3K27me3), peak-associated genes were compiled into gene sets for downstream comparison.

Differentially expressed genes (DEGs) identified by DESeq2 (false discovery rate [FDR] < 0.05) were intersected with peak-associated gene sets to identify genes exhibiting coordinated changes in chromatin state and transcription. Overlap significance between DEG lists and ChIP-associated gene sets was evaluated using a hypergeometric test, with *p*-values adjusted for multiple comparisons using the Benjamini–Hochberg procedure (FDR < 0.05 considered significant).

To assess the relationship between histone modification enrichment and transcriptional output, normalized ChIP-seq signal intensity (e.g., reads per kilobase per million mapped reads, RPKM, or normalized tag counts) within promoter regions was correlated with corresponding gene expression values obtained from RNA-seq. Correlation analyses were performed using Pearson correlation analysis. Statistical significance was defined as *p* < 0.05 after multiple-testing correction (Benjamini–Hochberg FDR).

Gene sets derived from integrated ChIP-seq and RNA-seq analyses were subjected to functional enrichment analysis using clusterProfiler. Gene Ontology (GO) and KEGG pathway enrichment were performed using a hypergeometric framework, with significance defined as adjusted *p*-values (FDR < 0.05). For visualization, integrated datasets were used to generate heatmaps, genomic signal profiles, and peak-to-gene association plots. BigWig files were visualized using genome browsers (e.g., IGV) to confirm concordance between histone modification enrichment and transcriptional activity at representative loci.

## 4. Conclusions

These findings characterize the epigenetic landscape of H3K27ac, H3K27me3, H3K4me1, and H3K4me3 following β-glucan exposure in channel catfish and suggest that remodeling of histone modifications contributes to transcriptional and metabolic reprogramming consistent with trained immunity. Integration of epigenetic and transcriptomic analyses indicates that β-glucan reshapes chromatin at immune-relevant loci, influencing gene regulation in pathways related to metabolism, signaling, pathogen recognition, and cellular remodeling. Pathway enrichment analyses highlighted key innate immune pathways, including Toll-like receptor, RIG-I-like receptor, phagocytosis, and apoptosis, supporting enhanced immune responsiveness. Gene-level changes, in factors such as *fadd*, *gnai2b*, *plcb4*, *itgb1b*, *rac1*, *tlr9*, *colec11*, and *phosphomannomutase*, further reflect coordinated regulation of immune signaling and cellular processes. However, not all histone modifications corresponded directly with gene expression, underscoring the complexity of chromatin–transcription relationships. Collectively, these results provide insight into how β-glucan may modulate innate immune responses in teleost fish. While consistent with features of trained immunity, further studies incorporating functional validation, time-course analyses, and cell-type-specific approaches are needed to better define the mechanisms and persistence of these effects and to support applications in aquaculture. Species-specific validation assays, such as Western blotting or ChIP-qPCR, to further confirm antibody specificity, should be included as well.

## Figures and Tables

**Table 1:** Gene set enrichment analysis (GSEA) details for the GO term (TRANSCRIPTION_FACTOR_BINDING (GO:0008134).

	GENE ID	GENE NAME (from Dataset)	RANK IN GENE LIST	RANK SCORE	RUNNING ES	CORE ENRICH.
1	ENSIPUG00000022770	crtc3	367	0.736703992	0.16947582	Yes
2	ENSIPUG00000005461	crtc1b	1561	0.502132237	0.24742988	Yes
3	ENSIPUG00000008205	ncoa2	2807	0.40567264	0.2992081	Yes
4	ENSIPUG00000023848	ncoa3	2857	0.403261602	0.3980275	Yes
5	ENSIPUG00000000346	-	3208	0.383159488	0.479833	Yes
6	ENSIPUG00000003445	-	4642	0.305455595	0.49907875	Yes
7	ENSIPUG00000001587	foxa3	5217	0.277373135	0.54552597	Yes
8	ENSIPUG00000004898	ncoa1	5426	0.268909633	0.6044382	Yes
9	ENSIPUG00000016979	gtf2b	11,295	0.02159046	0.37607652	No
10	ENSIPUG00000001537	foxa1	12,936	0	0.3107457	No
11	ENSIPUG00000024358	foxa2	13,272	0	0.29740068	No
12	ENSIPUG00000023313	brf1a	15,872	−0.016286094	0.19793698	No
13	ENSIPUG00000001075	-	17,110	−0.070176646	0.1661965	No
14	ENSIPUG00000016983	NCOA3	17,580	−0.09211456	0.17053206	No
15	ENSIPUG00000017884	brf1b	23,611	−0.518911421	0.05999295	No

## Data Availability

The data supporting the findings of this study have been deposited in Zenodo (DOI: 10.5281/zenodo.21229683) and are publicly available.

## Supplementary Materials

The following supporting information can be downloaded at: https://www.mdpi.com/article/10.3390/ijms27146282/s1.

## References

[1] Jaiswal A., Osborne T., Jaiswal A., Osborne T. (2026). Key Role of Histone Modifiers in Coupling Metabolism to Epigenetic Reprogramming Associated with “Trained Immunity” in Innate Immune Cells. Gene Expression-From Code to Cure.

[2] Fanucchi S., Domínguez-Andrés J., Joosten L.A.B., Netea M.G., Mhlanga M.M. (2021). The Intersection of Epigenetics and Metabolism in Trained Immunity. Immunity.

[3] Yan I., Guo H., Hu B., Li R., Yong J., Zhao Y., Zhi X., Fan X., Guo F., Wang X. (2016). Epigenomic Landscape of Human Fetal Brain, Heart, and Liver. J. Biol. Chem..

[4] Trovato M., Bunina D., Yildiz U., Fernandez-Novel Marx N., Uckelmann M., Levina V., Perez Y., Janeva A., Garcia B.A., Davidovich C. (2024). Histone H3.3 Lysine 9 and 27 Control Repressive Chromatin at Cryptic Enhancers and Bivalent Promoters. Nat. Commun..

[5] Bernstein B.E., Kamal M., Lindblad-Toh K., Bekiranov S., Bailey D.K., Huebert D.J., McMahon S., Karlsson E.K., Kulbokas E.J., Gingeras T.R. (2005). Genomic Maps and Comparative Analysis of Histone Modifications in Human and Mouse. Cell.

[6] Wang Z., Ren B. (2024). Role of H3K4 Mono-Methylation in Gene Regulation. Curr. Opin. Genet. Dev..

[7] Netea M.G., Joosten L.A.B., Latz E., Mills K.H.G., Natoli G., Stunnenberg H.G., O’Neill L.A.J., Xavier R.J. (2016). Trained Immunity: A Program of Innate Immune Memory in Health and Disease. Science.

[8] Petrie-Hanson L., Peterman A.E. (2022). Trained Immunity Provides Long-Term Protection against Bacterial Infections in Channel Catfish. Pathogens.

[9] Vetvicka V., Sima P., Vannucci L. (2021). Trained Immunity as an Adaptive Branch of Innate Immunity. Int. J. Mol. Sci..

[10] Pietras E.M., Reynaud D., Kang Y.A., Carlin D., Calero-Nieto F.J., Leavitt A.D., Stuart J.A., Göttgens B., Passegué E. (2015). Functionally Distinct Subsets of Lineage-Biased Multipotent Progenitors Control Blood Production in Normal and Regenerative Conditions. Cell Stem Cell.

[11] Netea M.G., Domínguez-Andrés J., Barreiro L.B., Chavakis T., Divangahi M., Fuchs E., Joosten L.A.B., van der Meer J.W.M., Mhlanga M.M., Mulder W.J.M. (2020). Defining Trained Immunity and Its Role in Health and Disease. Nat. Rev. Immunol..

[12] Meena D.K., Das P., Kumar S., Mandal S.C., Prusty A.K., Singh S.K., Akhtar M.S., Behera B.K., Kumar K., Pal A.K. (2013). Beta-Glucan: An Ideal Immunostimulant in Aquaculture (a Review). Fish Physiol. Biochem..

[13] Legentil L., Paris F., Ballet C., Trouvelot S., Daire X., Vetvicka V., Ferrières V. (2015). Molecular Interactions of β-(1→3)-Glucans with Their Receptors. Molecules.

[14] Soltanian S., Stuyven E., Cox E., Sorgeloos P., Bossier P. (2009). Beta-Glucans as Immunostimulant in Vertebrates and Invertebrates. Crit. Rev. Microbiol..

[15] Petit J., Bailey E.C., Wheeler R.T., Oliveira C.A.F.D., Forlenza M., Wiegertjes G.F. (2019). Studies into β-Glucan Recognition in Fish Suggests a Key Role for the C-Type Lectin Pathway. Front. Immunol..

[16] Rodrigues M.V., Zanuzzo F.S., Koch J.F.A., de Oliveira C.A.F., Sima P., Vetvicka V. (2020). Development of Fish Immunity and the Role of β-Glucan in Immune Responses. Molecules.

[17] Kraft K., Yosta K.E., Murphy S.E., Magg A., Long Y., Ryan Corces M., Granja J.M., Wittler L., Mundlos S., Cech T.R. (2022). Polycomb-Mediated Genome Architecture Enables Long-Range Spreading of H3K27 Methylation. Proc. Natl. Acad. Sci. USA.

[18] Bae S., Lesch B.J. (2020). H3K4me1 Distribution Predicts Transcription State and Poising at Promoters. Front. Cell Dev. Biol..

[19] Ma W., Noble W.S., Bailey T.L. (2014). Motif-Based Analysis of Large Nucleotide Data Sets Using MEME-ChIP. Nat. Protoc..

[20] Mulero M.C., Wang V.Y.F., Huxford T., Ghosh G. (2019). Genome Reading by the NF-ΚB Transcription Factors. Nucleic Acids Res..

[21] Csumita M., Csermely A., Horvath A., Nagy G., Monori F., Göczi L., Orbea H.A., Reith W., Széles L. (2019). Specific Enhancer Selection by IRF3, IRF5 and IRF9 Is Determined by ISRE Half-Sites, 5′ and 3′ Flanking Bases, Collaborating Transcription Factors and the Chromatin Environment in a Combinatorial Fashion. Nucleic Acids Res..

[22] Perini G., Diolaiti D., Porro A., Della Valle G. (2005). In Vivo Transcriptional Regulation of N-Myc Target Genes Is Controlled by E-Box Methylation. Proc. Natl. Acad. Sci. USA.

[23] Bogliotti Y.S., Ross P.J. (2012). Mechanisms of Histone H3 Lysine 27 Trimethylation Remodeling during Early Mammalian Development. Epigenetics.

[24] Aday A.W., Zhu L.J., Lakshmanan A., Wang J., Lawson N.D. (2011). Identification of Cis Regulatory Features in the Embryonic Zebrafish Genome through Large-Scale Profiling of H3K4me1 and H3K4me3 Binding Sites. Dev. Biol..

[25] Uengwetwanit T., Uawisetwathana U., Angthong P., Phanthura M., Phromson M., Tala S., Thepsuwan T., Chaiyapechara S., Prathumpai W., Rungrassamee W. (2025). Investigating a Novel β-Glucan Source to Enhance Disease Resistance in Pacific White Shrimp (Penaeus Vannamei). Sci. Rep..

[26] Bose N., Wurst L.R., Chan A.S.H., Dudney C.M., Leroux M.L., Danielson M.E., Will P.M., Nodland S.E., Patchen M.L., Dalle Lucca J.J. (2014). Differential Regulation of Oxidative Burst by Distinct β-Glucan-Binding Receptors and Signaling Pathways in Human Peripheral Blood Mononuclear Cells. Glycobiology.

[27] Rehman S., Gora A.H., Siriyappagouder P., Brugman S., Fernandes J.M.O., Dias J., Kiron V. (2021). Zebrafish Intestinal Transcriptome Highlights Subdued Inflammatory Responses to Dietary Soya Bean and Efficacy of Yeast β-Glucan. J. Fish Dis..

[28] Jacomin A.C., Dikic I. (2024). Membrane Remodeling via Ubiquitin-Mediated Pathways. Cell Chem. Biol..

[29] Tian M., Li X., Yu L., Qian J.X., Bai X.Y., Yang J., Deng R.J., Lu C., Zhao H., Liu Y. (2025). Glycosylation as an Intricate Post-Translational Modification Process Takes Part in Glycoproteins Related Immunity. Cell Commun. Signal..

[30] Beacon T.H., Delcuve G.P., López C., Nardocci G., Kovalchuk I., van Wijnen A.J., Davie J.R. (2021). The Dynamic Broad Epigenetic (H3K4me3, H3K27ac) Domain as a Mark of Essential Genes. Clin. Epigenet..

[31] Ferreira A.V., Domínguez-Andrés J., Merlo Pich L.M., Joosten L.A.B., Netea M.G. (2024). Metabolic Regulation in the Induction of Trained Immunity. Semin. Immunopathol..

[32] Altarejos J.Y., Montminy M. (2011). CREB and the CRTC Co-Activators: Sensors for Hormonal and Metabolic Signals. Nat. Rev. Mol. Cell Biol..

[33] Yu C., Li X., Zhao Y., Hu Y. (2023). The Role of FOXA Family Transcription Factors in Glucolipid Metabolism and NAFLD. Front. Endocrinol..

[34] Ranjan K., Pathak C. (2024). Cellular Dynamics of Fas-Associated Death Domain in the Regulation of Cancer and Inflammation. Int. J. Mol. Sci..

[35] Wang N., Li Y., Zhou X., Wang X., Yang G. (2023). Comprehensive Analysis Identifies ARHGEF6 as a Potential Prognostic and Immunological Biomarker in Lung Adenocarcinoma. Comput. Biol. Med..

[36] Ogata H., Goto S., Sato K., Fujibuchi W., Bono H., Kanehisa M. (1999). KEGG: Kyoto Encyclopedia of Genes and Genomes. Nucleic Acids Res..

[37] Kaukonen D., Kaukonen R., Polit L., Hennessy B.T., Lund R., Madden S.F. (2020). Analysis of H3K4me3 and H3K27me3 Bivalent Promotors in HER2+ Breast Cancer Cell Lines Reveals Variations Depending on Estrogen Receptor Status and Significantly Correlates with Gene Expression. BMC Med. Genom..

[38] Zhang Q., Zhang S., Chen J., Xie Z. (2023). The Interplay between Integrins and Immune Cells as a Regulator in Cancer Immunology. Int. J. Mol. Sci..

[39] Kumar D., Cinghu S., Oldfield A.J., Yang P., Jothi R. (2021). Decoding the Function of Bivalent Chromatin in Development and Disease. Genome Res..

[40] Liu G., Fang Y., Li J., Chen Z. (2025). Anoikis-Related PLCB4 Is Linked to Immunotherapy Response in Osteosarcoma. Discov. Oncol..

[41] Asahara S., Shibutani Y., Teruyama K., Inoue H.Y., Kawada Y., Etoh H., Matsuda T., Kimura-Koyanagi M., Hashimoto N., Sakahara M. (2013). Ras-Related C3 Botulinum Toxin Substrate 1 (RAC1) Regulates Glucose-Stimulated Insulin Secretion via Modulation of F-Actin. Diabetologia.

[42] Bekkering S., Arts R.J.W., Novakovic B., Kourtzelis I., van der Heijden C.D.C.C., Li Y., Popa C.D., ter Horst R., van Tuijl J., Netea-Maier R.T. (2018). Metabolic Induction of Trained Immunity through the Mevalonate Pathway. Cell.

[43] Lee G., Ahn H., Lee E., Lee G.S. (2023). The Role of NLRP3 Inflammasomes in Trained Immunity. Front. Biosci.-Landmark.

[44] Tamura Y., Yoneda A., Takei N., Sawada K. (2016). Spatiotemporal Regulation of Hsp90–Ligand Complex Leads to Immune Activation. Front. Immunol..

[45] Freeze H.H., Chong J.X., Bamshad M.J., Ng B.G. (2014). Solving Glycosylation Disorders: Fundamental Approaches Reveal Complicated Pathways. Am. J. Hum. Genet..

[46] Hajishengallis G., Li X., Mitroulis I., Chavakis T. (2019). Trained Innate Immunity and Its Implications for Mucosal Immunity and Inflammation. Adv. Exp. Med. Biol..

[47] Chaplin D.D. (2010). Overview of the Immune Response. J. Allergy Clin. Immunol..

[48] Yuan H., Gao Z., Lu X., Hu F. (2020). Role of Collectin-11 in Innate Defence against Uropathogenic Escherichia coli Infection. Innate Immun..

[49] Owen A.M., Fults J.B., Patil N.K., Hernandez A., Bohannon J.K. (2021). TLR Agonists as Mediators of Trained Immunity: Mechanistic Insight and Immunotherapeutic Potential to Combat Infection. Front. Immunol..

[50] Sanjabi S., Oh S.A., Li M.O. (2017). Regulation of the Immune Response by TGF-β: From Conception to Autoimmunity and Infection. Cold Spring Harb. Perspect. Biol..

[51] Dong X., Tsuji J., Labadorf A., Roussos P., Chen J.F., Myers R.H., Akbarian S., Weng Z. (2015). The Role of H3K4me3 in Transcriptional Regulation Is Altered in Huntington’s Disease. PLoS ONE.

[52] Sonnweber T., Pizzini A., Nairz M., Weiss G., Tancevski I. (2018). Arachidonic Acid Metabolites in Cardiovascular and Metabolic Diseases. Int. J. Mol. Sci..

[53] Tang X., Brindley D.N. (2020). Lipid Phosphate Phosphatases and Cancer. Biomolecules.

[54] Thompson L.L., Rutherford K.A., Lepage C.C., McManus K.J. (2021). The SCF Complex Is Essential to Maintain Genome and Chromosome Stability. Int. J. Mol. Sci..

[55] Milne T.A., Zhao K., Hess J.L. (2009). Chromatin Immunoprecipitation (ChIP) for Analysis of Histone Modifications and Chromatin-Associated Proteins. Methods Mol. Biol..

