# Supplementary material for: Epigenetic Landscape of H3K27ac, H3K27me3, H3K4me1, and H3K4me3 Marks in Channel Catfish Following Βeta Glucan Exposure
Source: Int J Mol Sci. 2026 Jul 15;27(14):6282. doi: 10.3390/ijms27146282 (PMC13410344; doi:10.3390/ijms27146282)
Supplement: Supplementary file 1 [file ijms-27-06282-s001.zip › ijms-4313278-supplementary.pdf]

Supplemental Table S1. Genes Associated with Differential H3K27ac and H3K27me3 Peaks and Their Genomic Annotations Identified by ChIP-Seq Analysis. Includes gene identifiers, peak direction, chromosomal location, exon count, strand orientation and gene size.

NCBI Gene ID	Ensemble Gene ID	Histone	Gene Name	peak	Chro	Exon count	Strand	Start	End	Gene size (BP)
562317	ENSDARG00000103021	H3K27ac	fadd	up	7	2	reverse	54312857	54319452	6,596
562640	ENSDARG00000002840	H3K27ac	si:dkey-28b4.8	up	1	23	reverse	44651727	44703898	52,172
558288	ENSDARG00000006683	H3K27ac	arhgef6	up	14	21	reverse	31799786	31854870	55,085
393968	ENSDARG00000103845	H3K27ac	Hnrpc or zgc:55733	down	7	10	reverse	4088029	4125021	36,993
559959	ENSDARG00000071699	H3K27me3	iduronate 2-sulfatase (ids) or zgc:158245	up	14	8	forward	20869635	20891746	22112
266599	NSDARG00000055283	H3K27me3	inhibitor of DNA binding 2a or id2; cb321; wu:fa04e03	up	17	3	forward	34805939	34808165	2,227
338204	ENSDARG00000042835	H3K27me3	transcription factor Dp-2, or id:ibd2511; wu:fb94f09; zgc:110490	up	15	13	forward	4421801	4491822	70022
100537538	ENSDARG00000100786	H3K27me3	plcb4a, phospholipase C, beta 4a, or plcb4, plcba	down	17	37	reverse	3704654	3829829	125176
793862	ENSDARG00000096549	H3K27me3	ras-related C3 botulinum toxin substrate 1 pseudogene, rac1	down	1		reverse	57128552	57129179	628
30265	ENSDARG00000014571	H3K27me3	catenin (cadherin-associated protein), beta 1 (ctnnb1) or ctnnb; id:ibd2058; wu:fb73e10; wu:fi81c06; wu:fk25h01	down	16	19	forward	6182500	6249214	66715
118437	ENSDARG00000015472	H3K27me3	gpc4 glypican 4 0r kny; gpc6; gpc4,; gpc4/6; knypek; fc47a08; fe05f10; chunp6920; wu:fc47a08; wu:fe05f10; zgc:194854	down	14	9	reverse	31092327	31151148	58822
556272	ENSDARG00000075129	H3K27me3	mrps11, sb:eu1637; zgc:162401; si:dkey-265m8.2	down	7	6	reverse	14511993	14535888	23896
393818	ENSDARG00000104011	H3K27me3	rps17, ribosomal protein S17, zgc:77702	down	7	5	reverse	54092524	54098566	6043
558312	ENSDARG00000011201	H3K27me3	rplp2l ribosomal protein, large P2, like	down	7	5	reverse	12959846	12968698	8853
569581	ENSDARG00000060554	H3K27me3	methylmalonyl CoA mutase, mmut, mut	down	20	13	reverse	44372745	44460789	88045
394190	ENSDARG00000021220	H3K27me3	3-hydroxy-3-methylglutaryl-CoA lyase, hmgcl, zgc:56248	down	17	18	reverse	24855678	24866727	11050
	ENSDARG00000100267	H3K27me3		down			reverse			
567820	ENSDARG00000005522	H3K27me3	galr1a galanin receptor 1a, galr1; si:ch211-150o20.3	down	19	3	forward	21919912	0.21924148	4237

100331465	ENSDARG00000076824	H3K27me3	cholecystokinin B receptor b, cckbrb, cckrl; cckr-like	down	9	5	reverse	5775100	5856433	81334
101882850	ENSDARG00000061940	H3K27me3	5-hydroxytryptamine receptor 4, htr4, si:ch211-228b4.1	down	14	8	reverse	25127822	25309142	181321
793862	ENSDARG00000096549	H3K27me3	ras-related C3 botulinum toxin substrate 1 pseudogene, rac1	down	1		reverse	57128552	57129179	628
570216	ENSDARG00000104484	H3K27me3	integrin, beta 1b, itgb1b, integrin, beta 1b	down	2	9	reverse	43577293	43583881	6589
565155	ENSDARG00000024746	H3K27me3	heat shock protein 90, alpha (cytosolic), class A member 1, tandem duplicate 2hsp90aa1.2, cb820, hsp90a.2, hsp90a2	down	20	11	reverse	53967596	53981618	14023
100005885	ENSDARG00000059131	H3K27me3	speedy/RINGO cell cycle regulator family member A spdya, zgc:101624	down	17	7	forward	24809223	24815220	5,998
337077	ENSDARG00000100562	H3K27me3	syndecan 3, sdc3, wu:fa97a01	down	un	4	forward	4451	35663	31213
394190	ENSDARG00000021220	H3K27me3	3-hydroxy-3-methylglutaryl-CoA lyase, hmgcl, zgc:56248	down	17	8	reverse	24855678	24866727	11,050
100536353	ENSDARG00000074556	H3K27me3	glucosidase, alpha; neutral C, ganc, si:ch73-70a17.1	down	17	25	forward	45259267	45302903	43,637
280652	ENSDARG00000061328	H3K27me3	cell adhesion associated, oncogene regulated, cdon, cb507; wu:fa10c03; wu:fb94b11; zgc:158240	down	18	19	reverse	44053013	44129028	76016
114426	ENSDARG00000007377	H3K27me3	ornithine decarboxylase 1, odc1, CHUNP6922, fc54f04, fi06d08, odc, wu:fc54f04, wu:fi06d08	down	17	11	forward	51743921	51757548	13628
553594	ENSDARG00000008890	H3K27me3	cystinosin, lysosomal cystine transporter, ctns, zgc:110194	down	11	10	forward	6281647	6293709	12063
100004503	ENSDARG00000069951	H3K27me3	eukaryotic translation elongation factor 1 alpha 1b, eef1a1b, eef1a1; wu:fj34g08; zgc:110335	down	1	8	forward	31112436	31130222	17787
791973	ENSDARG00000041041	H3K27me3	chemokine (C-X-C motif) receptor 3, tandem duplicate 2, cxcr3.2, zgc:92301	down	16	3	forward	11818136	11825303	7168

Supplemental Table S2. Genes Associated with Differential H3K4me1 and H3K4me3 Peaks and Their Genomic Annotations Identified by ChIP-Seq Analysis. Includes gene identifiers, peak direction, chromosomal location, exon count, strand orientation and gene size.

NCBI Gene ID	Ensemble Gene ID	Histone	Gene Name	peak	Chro	Exon count	Strand	Start	End	Gene size (BP)
550552	ENSDARG00000099430	H3K4me1	ATG16 autophagy related 16-like 1, atg16l1, fb57e05; wu:fb57e05; zgc:110147	up	15	20	forward	45591663	45637461)	45798
100331333	ENSDARG00000103056	H3K4me1	integrin alpha 4, itga4, si:ch73-119a20.1	up	9	29	forward	44034927	44088380	53454
30671	ENSDARG00000021924	H3K4me1	heat shock cognate 70-kd protein, tandem duplicate 3, hsp70; hspa1a; hsp70-4, hsp70.3	up	3	2	forward	26123736	26127091	3356
562014	ENSDARG00000055470	H3K4me1	cell division cycle 16 homolog (S. cerevisiae), cdc16, zgc:123264	down	9	18	reverse	34920001	34937229	17229
569575	ENSDARG00000035966	H3K4me1	progesterone receptor, pgr, pg; pr; gb:dq017620	down	18	8	forward	41819793	41839720	19,928
327650	ENSDARG00000017294	H3K4me1	guanine nucleotide binding protein (G protein), alpha inhibiting activity polypeptide 2b, gnai2; fi21e06; zgc:56690; wu:fi21e06, gnai2b	down	6	9	reverse	53050166	53143701	93536
560649	ENSDARG00000096445	H3K4me1	phospholipase B1, plb1, si:ch211-214p16.3	down	17	42	reverse	25649940	25670533	20594
557680	ENSDARG00000059933	H3K4me1	phospholipid phosphatase 3, plpp3, lpp3; ppap2b; si:dkey-19f4.1	down	20	7	reverse	7965217	8024297	59,081
566831	ENSDARG00000035655	H3K4me1	RAD21 cohesin complex component b, rad21b, si:ch211-246f9.1	down	19	14	reverse	44068001	44091381	23381
58092	ENSDARG00000036096	H3K4me1	SMAD family member 3a, smad3a, madh3; smad3; madh3a; wu:fa99e03	down	7	9	reverse	34106111	34149234	43124
65230	ENSDARG00000044062	H3K4me1	C-terminal binding protein 2a, ctbp2a, ctbp2; fe05b01; wu:fb96g03; wu:fe05b01; wu:fk19e02	down	17	14	reverse	8596943	8727731	95840
563306	ENSDARG00000052091	H3K4me1	recombination signal binding protein for immunoglobulin kappa J region b, rbpjb, Su(H)2; su(h)B	down	7	11	forward	62080512	62206922	126411
406255	ENSDARG00000007885	H3K4me1	FA complementation group L, fancl, zgc:66144; zgc:76938	down	13	14	forward	26703897	26780798	76902

100333154	ENSDARG00000076111	H3K4me1	glucoside xylosyltransferase 1a, gxylt1a	down	25	6	reverse	265337	269559	4223
447818	ENSDARG00000102998	H3K4me1	TAF6 RNA polymerase II, TATA box binding protein (TBP)-associated factor, taf6, zgc:92160; wu:fb83e08; wu:fj30b02	down	5	16	forward	53581485	53761950	180,466
64610	ENSDARG00000018259	H3K4me1	ATPase Na+/K+ transporting subunit alpha 3a, atp1a3a, cb705; atp[a]3B; wu:fj56a06	down	19	21	reverse	6328245	6385541	57297
100007686	ENSDARG00000038398	H3K4me1	phosphomannomutase 1, LOC100007686	down	3	6	forward	3810708	3821783	11076
100005717	ENSDARG00000038618	H3K4me1	carnitine palmitoyltransferase 2, cpt2, wu:fa03e08; wu:fb54a02; zgc:101627	down	8	6	reverse	21137453	21142599	5110
562292	ENSDARG00000059503	H3K4me1	acyl-CoA synthetase family member 3, acsf3,	down	7	11	forward	55950294	56037907	87614
556956	ENSDARG00000076724	H3K4me1	phosphatidylinositol 4-kinase, catalytic, alpha a, pi4kaa, pi4ka	down	10	55	reverse	3186312	0.3261158	74847
406788	ENSDARG00000020043	H3K4me1	CCR4-NOT transcription complex, subunit 8, cnot8, zgc:63844; wu:fe49a05	down	21	7	reverse	36442084	36453451	11368
100038774	ENSDARG00000042521	H3K4me1	si:dkey-251i10.1	down	5	4	forward	67448514	67462334	13821
30509	ENSDARG00000110398	H3K4me1	opioid receptor, delta 1a, ZFOR1; oprd1, oprd1a	down	19	3	forward	14920968	14951756	30789
494163	ENSDARG00000021509	H3K4me3	TGF-beta activated kinase 1 (MAP3K7) binding protein 2, tab2, map3k7ip2; zgc:77446	up	20	8	forward	1329523	1350171	20649
563727	ENSDARG00000061710	H3K4me3	mitogen-activated protein kinase kinase kinase 8, map3k8,	up	12	8	reverse	23645755	23658915	13161
403128	ENSDARG00000044490	H3K4me3	toll-like receptor 9, tlr9, sb:eu392	up	8	1	reverse	53730368	53733659	3292
402862	ENSDARG00000029018	H3K4me3	cdkn1ba, cyclin dependent kinase inhibitor 1Ba, cdkn1bl	up	4	3	reverse	18592743	18595551	2,809
327204	ENSDARG00000074849	H3K4me3	Rac family small GTPase 1a, rac1a, rac1; zgc:55823; zgc:55917; zgc:86934; wu:fd16e02	up	12	6	reverse	10464897	10476337	11441
369194	ENSDARG00000027087	H3K4me3	transforming growth factor, beta 2, tgfb2	up	16	8	reverse	2743381	2808096	62,522
492459	ENSDARG00000013266	H3K4me3	collectin sub-family member 11, colec11, wu:fb82e09; zgc:103572	up	17	11	reverse	36516697	36675721	159025
335110	ENSDARG00000113183	H3K4me3	dynein, cytoplasmic 1, heavy chain 1, dync1h1, fk70a07; wu:fk70a07	up	17	73	reverse	871205	945956	74,752
393441	ENSDARG00000016038	H3K4me3	3-hydroxyacyl-CoA dehydratase 3, hacd3, HACD; ptplad1; zgc:63632	up	18	11	forward	18982077	18995283	13207
336944	ENSDARG00000038968	H3K4me3	chemokine (C-C motif) receptor 6b, ccr6b, wu:fk31f08	up	17	2	reverse	51642761	51651607	8,847

336526	ENSDARG00000018971	H3K4me3	UDP-GlcNAc:betaGal beta-1,3-N-acetylglucosaminyltransferase 5a, b3gnt5a, Lc3; b3gnt5; fb12f12; wu:fb12f12; wu:fe50g08	up	11	2	forward	10541258	10550309	9,052
394103	ENSDARG00000058102	H3K4me3	sarcosine dehydrogenase, sardh, zgc:56363	up	10	22	forward	10386571	10561822	175251
393261	ENSDARG00000070674	H3K4me3	proteasome 26S subunit, non-ATPase 6, psmd6, zgc:56471	up	11	8	forward	19502367	19511548	9182
553418	ENSDARG00000067829	H3K4me3	peroxisome proliferator-activated receptor gamma, coactivator 1 alpha, ppargc1a, PGC1; PGC-1; ppargc1al; PGC-1alpha; gb:dq017637	up	7	13	reverse	70575025	70669518	94,494
100330031	ENSDARG00000099199	H3K4me3	itchy E3 ubiquitin protein ligase b, itchb, itch; itcha	up	23	24	reverse	43531956	43595780	63825
101885491	ENSDARG00000099109	H3K4me3	multiple PDZ domain crumbs cell polarity complex component, mpdz	up	7	48	reverse	72641074	72748561	107488
559087	ENSDARG00000017141	H3K4me3	phospholipase A2, group IVAb (cytosolic, calcium-dependent), pla2g4ab, pla2g4a; si:dkey-97o5.1	down	20	18	forward	34128597	34144908	16,312
569053	ENSDARG00000053381	H3K4me3	phospholipid phosphatase 1a, plpp1a, lpp1; ppap2a; im:7157400; si:ch73-96j23.1	down	10	7	reverse	8265972	8295329	29,358
450083	ENSDARG00000036155	H3K4me3	galactosidase, alpha, gla, zgc:101584	down	14	7	reverse	38932445	38946799	14355
553750	ENSDARG00000056690	H3K4me3	myotubularin related protein 1a, mtmr1a, mtmr1; zgc:113282	down	7	18	forward	25858394	25877684	19291
326954	ENSDARG00000025904	H3K4me3	trans-2,3-enoyl-CoA reductase b, tecrb, cb250, gpsn2, mg:db03b10, sb:cb250, wu:fj63b12	down	1	12	forward	55752643	55779096	24512
393984	ENSDARG00000012468	H3K4me3	acetoacetyl-CoA synthetase, aacs, zgc:56105	down	5	18	reverse	18825167	18897555	72389
335629	ENSDARG00000101406	H3K4me3	ribosomal protein, large P2, rplp2, RpP1; zgc:73372; wu:fb05g05; wu:fj30h02	down	6	5	reverse	49508800	49511576	2777
570613	ENSDARG00000053405	H3K4me3	sorbitol dehydrogenase, sord, zgc:63674	down	7	9	reverse	30923283	30926009	2,727
558326	ENSDARG00000061454	H3K4me3	neurexin 2a, nrxn2a,	down	21	25	reverse	27635485	28340997	705,513
	ENSDARG00000098523	H3K4me3		down						
571246	ENSDARG00000059616	H3K4me3	heparan sulfate (glucosamine) 3-O-sulfotransferase 2, hs3st2	down	3	2	forward	44947355	45000645	53291
556315	ENSDARG00000022550	H3K4me3	glucoside xylosyltransferase 1b, gxylt1b, glt8d3; gxylt1; si:ch211-155a11.6	down	4	7	reverse	13893268	13902148	8881
798657	ENSDARG00000086778	H3K4me3	platelet-derived growth factor beta polypeptide a, pdgfba, pdgfb; sb:eu1033; si:ch211-79m20.1; si:dkey-261d11.1,	down	22	7	reverse	29309393	29336398	27,006
100001424	ENSDARG00000054771	H3K4me3	growth hormone receptor a, ghra, ghr; ghr.a; zgc:162141	down	8	9	reverse	31465222	31606409	141188
393716	ENSDARG00000003151	H3K4me3	S-phase kinase-associated protein 1, skp1, skp1a; zgc:73186	down	21	6	reverse	45341786	45349486	7701

368488	ENSDARG00000044972	H3K4me3	B cell receptor associated protein 31, bcap31, si:bz30i22.4, si:rp71-30i22.4, zgc:56389	down	8	8	forward	8973425	9029176	55752
565910	ENSDARG00000070536	H3K4me3	creb5b, cAMP responsive element binding protein 5b, si:ch73-362g18.2	down	16	11	reverse	20529129	20707702	178574
572649	ENSDARG00000036135	H3K4me3	butyrobetaine (gamma), 2-oxoglutarate dioxygenase (gamma-butyrobetaine hydroxylase) 1, bbox1, wu:fj14g08; zgc:112179	down	7	9	forward	34296789	34315292	15646
	ENSDARG00000002956	H3K4me3		down						
393152	ENSDARG00000101543	H3K4me3	WASH complex subunit 5, washc5, zgc:55908; strumpellin	down	16	29	reverse	43291709	43337746	46,038
100009646	ENSDARG00000099240	H3K4me3	ArfGAP with coiled-coil, ankyrin repeat and PH domains 3b, acap3b, zgc:158388	down	11	27	reverse	3385104	3494964	109861
664765	ENSDARG00000069852	H3K4me3	lipoyl(octanoyl) transferase 2, lipt2, zgc:136925	down	15	4	reverse	4957780	4969258	11479
561906	ENSDARG00000017790	H3K4me3	cholinergic receptor, nicotinic, beta 2, chrnb2, chrnb2b	down	16	6	forward	22618323	22675645	57323

Supplemental Table S3. Chromatin Landscape Associated Differential Gene Expression and KEGG Pathway Enrichment One Month After β-Glucan Exposure. Includes peak direction, frequency of histone mark enrichment, and associated KEGG pathways ($P < 0.05$).

**Gene Name**	**peak**	**Number of expressed times**	**Pathways related DEGs**
Fadd	up	4 times H3K27ac and 4 times H3K4 me3	RIG-I-like receptor signaling pathway, Toll-like receptor signaling pathway, Apoptosis, and Herpes simplex infection
si:dkey-28b4	up	1 times H3K27ac	Calcium signaling pathway
arhgef6	up	1 times H3K27ac	Regulation of actin cytoskeleton
Hnrpc	down	1 times H3K27ac	Spliceosome
iduronate 2-sulfatase (IDS) or zgc:158245	up	3 times H3K27me3 and 3 times H3K4 me3	Lysosome, Metabolic pathways, and Glycosaminoglycan degradation
inhibitor of DNA binding 2a (ID2a)	up	1 times H3K27me3	TGF-beta signaling pathway
transcription factor Dp-2	up	1 times H3K27me3	Cell cycle
plcb4a, phospholipase C, beta 4a, or plcb4, plcba	down	11 times H3K27me3 and 11 times H3K4 me3	Wnt signaling pathway, Calcium signaling pathway, AGE-RAGE signaling pathway in diabetic complications, Inositol phosphate metabolism, Metabolic pathways, Melanogenesis, Phosphatidylinositol signaling system, GnRH signaling pathway, Gap junction, Vascular smooth muscle contraction, and Adrenergic signaling in cardiomyocytes
catenin (cadherin-associated protein), beta 1 (ctnnb1) or ctnnb; id:ibd2058; wu:fb73e10; wu:fi81c06; wu:fk25h01	down	5 times H3K27me3	Wnt signaling pathway, Adherens junction, Focal adhesion, Melanogenesis, and Tight junction
rps17, ribosomal protein S17, zgc:77702	down	1 times H3K27me3 and 1 times H3K4 me1	Ribosome
rplp2, ribosomal protein 2	down	1 times H3K27me3	Ribosome
methylmalonyl CoA mutase, mmut	down	1 times H3K27me3	Metabolic pathways
galr1a, galanin receptor 1a, galr1; si:ch211-150o20.3	down	1 times H3K27me3	Neuroactive ligand-receptor interaction
cholecystokinin B receptor b, cckbrb, cckrl; cckr-like	down	2 times H3K27me3	Calcium signaling pathway and Neuroactive ligand-receptor interaction
5-hydroxytryptamine receptor 4, htr4, si:ch211-228b4.1	down	2 times H3K27me3	Calcium signaling pathway and Neuroactive ligand-receptor interaction
integrin, beta 1b, itgb1b, integrin, beta 1b	down	5 times H3K27me3	Focal adhesion, Cell adhesion molecules, Regulation of actin cytoskeleton, ECM-receptor interaction, and Phagosome
heat shock protein 90, alpha (cytosolic), class A member 1, tandem duplicate 2 hsp90aa1.2, cb820, hsp90a.2, hsp90a2	down	3 times H3K27me3	Protein processing endoplasmic reticulum, NOD-like receptor signaling pathway, and Progesterone-mediated oocyte maturation
speedy/RINGO cell cycle regulator family member A spdya, zgc:101624	down	2 times H3K27me3 and 2 times H3K4 me1	Oocyte meiosis and Progesterone-mediated oocyte maturation

syndecan 3, sdc3, wu:fa97a01	down	1 times H3K27me3	Cell adhesion molecules
glucosidase, alpha; neutral C, ganc, si:ch73-70a17.1	down	3 times H3K27me3	Metabolic pathways, Starch and sucrose metabolism, and Galactose metabolism
cell adhesion associated, oncogene regulated, cdon, cb507; wu:fa10c03; wu:fb94b11; zgc:158240	down	1 times H3K27me3 and 1 times H3K4me1	Hedgehog signaling pathway
cystinosin, lysosomal cystine transporter, ctns, zgc:110194	down	1 times H3K27me3	Lysosome
eukaryotic translation elongation factor 1 alpha 1b, eef1a1b, eef1a1; wu:fj34g08; zgc:110335	down	1 times H3K27me3	RNA transport
ras-related C3 botulinum toxin substrate 1 pseudogene, rac1	down	5 times H3K27me3	Wnt signaling pathway, Toll-like receptor signaling pathway, MAPK signaling pathway, Phagosome, Regulation of actin cytoskeleton, and VEGF signaling pathway
gpc4 glypican 4 0r kny; gpc6; gpc4; gpc4/6; knypek; fc47a08; fe05f10; chunp6920; wu:fc47a08; wu:fe05f10; zgc:194854	down	1 times H3K27me3	Wnt signaling pathway
3-hydroxy-3-methylglutaryl-CoA lyase, hmgcl, zgc:56248	down	5 times H3K27me3	Valine, leucine and isoleucine degradation, Synthesis and degradation of ketone bodies, Butanoate metabolism, Peroxisome, and Metabolic pathways
ornithine decarboxylase 1, odc1, CHUNP6922, fc54f04, fi06d08, odc, wu:fc54f04, wu:fi06d08	down	3 times H3K27me3	Glutathione metabolism, Arginine and proline metabolism, Metabolic pathways, and Glutathione metabolism
Chemokine (C-X-C motif) receptor 3, tandem duplicate 2, cxcr3.2, zgc:92301	down	1 times H3K27me3	Cytokine-cytokine receptor interaction
ATG16 autophagy related 16-like 1, atg16l1, fb57e05; wu:fb57e05; zgc:110147	up	1 times H3K4me1	Regulation of autophagy
integrin alpha 4, itga4, si:ch73-119a20.1	up	5 times H3K4me1	Intestinal immune network for IgA production, ECM-receptor interaction, Cell adhesion molecules, Focal adhesion, and Regulation of actin cytoskeleton
heat shock cognate 70-kd protein, tandem duplicate 3, hsp70; hspa1a; hsp70-4, hsp70.3	up	4 times H3K4me1	Spliceosome, Protein processing in endoplasmic reticulum, MAPK signaling pathway, and Endocytosis
progesterone receptor, pgr, pg; pr; gb:dq 017620	down	2 times H3K4me1	Progesterone-mediated oocyte maturation and Oocyte meiosis

guanine nucleotide binding protein (G protein), alpha inhibiting activity polypeptide 2b, gnai2; fi21e06; zgc:56690; wu:fi21e06, gnai2b	down	5 times H3K4me1	Progesterone-mediated oocyte maturation, Adrenergic signaling in cardiomyocytes, Gap junction, Melanogenesis, and Tight junction
RAD21 cohesin complex component b, rad21b, si:ch211-246f9.1	down	1times H3K4me1	Cell cycle
C-terminal binding protein 2a, ctbp2a, ctbp2; fe05b01; wu:fb96g03; wu:fe05b01; wu:fk19e02	down	2 times H3K4me1	Notch signaling pathway and Wnt signaling pathway
FA complementation group L, fancl, zgc:66144; zgc:76938	down	2 times H3K4me1	Ubiquitin mediated proteolysis and Fanconi anemia pathway
glucoside xylosyltransferase 1a, gxylt1a	down	1 times H3K4me1	O-glycan biosynthesis
TAF6 RNA polymerase II, TATA box binding protein (TBP)-associated factor, taf6, zgc:92160; wu:fb83e08; wu:fj30b02	down	2 times H3K4me1	Basal transcription factors and Herpes simplex infection
phosphomannomutase 1, LOC100007686	down	3 times H3K4me1	Fructose and mannose metabolism, Amino sugar and nucleotide sugar metabolism, and Metabolic pathways
carnitine palmitoyltransferase 2, cpt2, wu:fa03e08; wu:fb54a02; zgc:101627	down	3 times H3K4me1	Fatty acid degradation, Fatty acid metabolism, and PPAR signaling pathway
phosphatidylinositol 4-kinase, catalytic, alpha a, pi4kaa, pi4ka	down	3 times H3K4me1	Inositol phosphate metabolism, Phosphatidylinositol signaling system, and Metabolic pathways
si:dkey-251i10.1	down	1 times H3K4me1	Calcium signaling pathway
opioid receptor, delta 1a, ZFOR1; oprd1, oprd1a	down	1 times H3K4me1	Neuroactive ligand-receptor interaction
cell division cycle 16 homolog (S. cerevisiae), cdc16, zgc:123264	down	4 times H3K4me1	Progesterone-mediated oocyte maturation, Oocyte meiosis, Cell cycle, and Ubiquitin mediated proteolysis
phospholipase B1, plb1, si:ch211-214p16.3	down	6 times H3K4me1	Ether lipid metabolism, Glycerophospholipid metabolism, alpha-Linolenic acid metabolism, Linoleic acid metabolism, Arachidonic acid metabolism, and Metabolic pathways
phospholipid phosphatase 3, plpp3, lpp3; ppap2b; si:dkey-19f4.1	down	5 times H3K4me1	Ether lipid metabolism, Glycerophospholipid metabolism, Sphingolipid metabolism, Glycerolipid metabolism, and Metabolic pathways

SMAD family member 3a, smad3a, madh3; smad3; madh3a; wu: fa99e03	down	6 times H3K4me1	Cell cycle, Adherens junction, TGF-beta signaling pathway, AGE-RAGE signaling pathway in diabetic complications, FoxO signaling pathway, and Endocytosis
recombination signal binding protein for immunoglobulin kappa J region b, rbpjb, Su(H)2; su(h)B	down	1time H3K4me1	Notch signaling pathway
ATPase Na+/K+ transporting subunit alpha 3a, atp1a3a, cb705; atp[a]3B; wu:fj56a06	down	2 times H3K4me1	Adrenergic signaling in cardiomyocytes and Cardiac muscle contraction
acyl-CoA synthetase family member 3, acsf3	down	3 times H3K4me1	Valine, leucine and isoleucine degradation
CCR4-NOT transcription complex, subunit 8, cnot8, zgc:63844; wu:fe49a05	down	1 times H3K4me1	RNA degradation
3-hydroxyacyl-CoA dehydratase 3, hacd3, HACD; ptplad1; zgc:63632	up	3 times H3K4me3	Biosynthesis of unsaturated fatty acids, Fatty acid elongation, Fatty acid metabolism, and Biosynthesis of unsaturated fatty acids
peroxisome proliferator-activated receptor gamma, coactivator 1 alpha, ppargc1a, PGC1; PGC-1; ppargc1al; PGC-1alpha; gb:dq017637	up	2 times H3K4me3	Adipocytokine signaling pathway and Insulin signaling pathway
itchy E3 ubiquitin protein ligase b, itchb, itch; itcha	up	2 times H3K4me3	Endocytosis and Ubiquitin mediated proteolysis
TGF-beta activated kinase 1 (MAP3K7) binding protein 2, tab2, map3k7ip2; zgc:77446	up	4 times H3K4me3	Toll-like receptor signaling pathway, MAPK signaling pathway, Herpes simplex infection, and NOD-like receptor signaling pathway
mitogen-activated protein kinase kinase kinase 8, map3k8	up	2 times H3K4me3	Toll-like receptor signaling pathway and MAPK signaling pathway
toll-like receptor 9, tlr9, sb:eu392	up	2 times H3K4me3	Toll-like receptor signaling pathway and Herpes simplex infection
cdkn1ba, cyclin dependent kinase inhibitor 1Ba, cdkn1bl	up	4 times H3K4me3	AGE-RAGE signaling pathway in diabetic complications, Cell cycle, FoxO signaling pathway, and ErbB signaling pathway
Rac family small GTPase 1a, rac1a, rac1; zgc:55823; zgc:55917; zgc:86934; wu:fd16e02	up	10 times H3K4me3	Toll-like receptor signaling pathway, AGE-RAGE signaling pathway in diabetic complications, Phagosome, MAPK signaling pathway, Salmonella infection, VEGF signaling pathway, Adherens junction, Wnt signaling pathway, Focal adhesion, and Regulation of actin cytoskeleton

transforming growth factor, beta 2, tgfb2	up	7 times H3K4me3	AGE-RAGE signaling pathway in diabetic complications, MAPK signaling pathway, Cell cycle, Cytokine-cytokine receptor interaction, FoxO signaling pathway, Endocytosis, and TGF-beta signaling pathway
collectin sub-family member 11, colec11, wu:fb82e09; zgc:103572	up	1 times H3K4me3	Phagosome
dynein, cytoplasmic 1, heavy chain 1, dync1h1, fk70a07; wu:fk70a07	up	2 times H3K4me3	Phagosome and Salmonella infection
chemokine (C-C motif) receptor 6b, ccr6b, wu:fk31f08	up	1times H3K4me3	Cytokine-cytokine receptor interaction
UDP-GlcNAc:betaGal beta-1,3-N-acetylglucosaminyltransferase 5a, b3gnt5a, Lc3; b3gnt5; fb12f12; wu:fb12f12; wu:fe50g08	up	2 times H3K4me3	Glycosphingolipid biosynthesis - lacto and neolacto series and Metabolic pathways
sarcosine dehydrogenase, sardh, zgc:56363	up	2 times H3K4me3	Metabolic pathways, Glycine, serine and threonine metabolism
proteasome 26S subunit, non-ATPase 6, psmd6, zgc:56471	up	1 times H3K4me3	Proteasome
multiple PDZ domain crumbs cell polarity complex component, mpdz	up	1 times H3K4me3	Tight junction
phospholipase A2, group IVAb (cytosolic, calcium-dependent), pla2g4ab, pla2g4a; si:dkey-97o5.1	down	10 times H3K4me3	Ether lipid metabolism, Glycerophospholipid metabolism, alpha-Linolenic acid metabolism, Linoleic acid metabolism, GnRH signaling pathway, Vascular smooth muscle contraction, Arachidonic acid metabolism, MAPK signaling pathway, VEGF signaling pathway, and Metabolic pathways
phospholipid phosphatase 1a, plpp1a, lpp1; ppap2a; im:7157400; si:ch73-96j23.1	down	5 times H3K4me3	Ether lipid metabolism, Glycerolipid metabolism, Sphingolipid metabolism, Glycerophospholipid metabolism, and Metabolic pathways
galactosidase alpha, gla, zgc:101584	down	5 times H3K4me3	Glycerolipid metabolism, Sphingolipid metabolism, Glycosphingolipid biosynthesis - globo series, and Galactose metabolism, Lysosome
Myotubularin related protein 1a, mtmr1a, mtmr1; zgc:113282	down	3 times H3K4me3	Inositol phosphate metabolism, Phosphatidylinositol signaling system, and Metabolic pathways
acetoacetyl-CoA synthetase, aacs, zgc:56105	down	2 times H3K4me3	Butanoate metabolism and

			Valine, leucine and isoleucine degradation
ribosomal protein, large P2, rplp2, RpP1; zgc:73372; wu:fb05g05; wu:fj30h02	down	1 times H3K4me3	Ribosome
sorbitol dehydrogenase, sord, zgc:63674	down	3 times H3K4me3	Pentose and glucuronate interconversions, Fructose and mannose metabolism, and Metabolic pathways
neurexin 2a, nrxn2a	down	1times H3K4me3	Cell adhesion molecules
heparan sulfate (glucosamine) 3-O-sulfotransferase 2, hs3st2	down	1times H3K4me3	Glycosaminoglycan biosynthesis - heparan sulfate / heparin
glucoside xylosyltransferase 1b, gxylt1b, glt8d3; gxylt1; si:ch211-155a11.6	down	1times H3K4me3	O-glycan biosynthesis
growth hormone receptor a, ghra, ghr; ghr.a; zgc:162141	down	2 times H3K4me3	Cytokine-cytokine receptor interaction and Neuroactive ligand-receptor interaction
S-phase kinase-associated protein 1, skp1, skp1a; zgc:73186	down	7 times H3K4me3	Wnt signaling pathway, Protein processing in endoplasmic reticulum, TGF-beta signaling pathway, Oocyte meiosis, Cell cycle, Ubiquitin mediated proteolysis, and Herpes simplex infection
creb5b, cAMP responsive element binding protein 5b, si:ch73-362g18.2	down	1 times H3K4me3	Adrenergic signaling in cardiomyocytes
butyrobetaine (gamma), 2-oxoglutarate dioxygenase (gamma-butyrobetaine hydroxylase) 1, bbox1, wu:fj14g08; zgc:112179	down	1 times H3K4me3	Lysine degradation
WASH complex subunit 5, washc5, zgc:55908; strumpellin	down	1 times H3K4me3	Endocytosis
cholinergic receptor, nicotinic, beta 2, chrnb2, chrnb2b	down	1 times H3K4me3	Neuroactive ligand-receptor interaction
trans-2,3-enoyl-CoA reductase b, tecrb, cb250, gpsn2, mg:db03b10, sb:cb250, wu:fj63b12	down	3 times H3K4me3	Biosynthesis of unsaturated fatty acids, Fatty acid elongation, and Fatty acid metabolism

platelet-derived growth factor beta polypeptide a, pdgfba, pdgfb; sb:eu1033; si:ch211-79m20.1; si:dkey-261d11.1,	down	4 times H3K4me3	Cytokine-cytokine receptor interaction, Focal adhesion, Regulation of actin cytoskeleton, and MAPK signaling pathway
B cell receptor associated protein 31, bcap31, si:bz30i22.4, si:rp71-30i22.4, zgc:56389	down	1 times H3K4me3	Protein processing in endoplasmic reticulum
ArfGAP with coiled-coil, ankyrin repeat and PH domains 3b, acap3b, zgc:158388	down	1 times H3K4me3	Endocytosis
lipoyl(octanoyl) transferase 2, lipt2, zgc:136925	down	1 times H3K4me3	Metabolic pathways
